# Circulating Tumour Cells (CTCs) in NSCLC: From Prognosis to Therapy Design

**DOI:** 10.3390/pharmaceutics13111879

**Published:** 2021-11-05

**Authors:** Zdeněk Kejík, Robert Kaplánek, Petr Dytrych, Michal Masařík, Kateřina Veselá, Nikita Abramenko, David Hoskovec, Martina Vašáková, Jarmila Králová, Pavel Martásek, Milan Jakubek

**Affiliations:** 1 Department of Paediatrics and Inherited Metabolic Disorders, First Faculty of Medicine, Charles University and General University Hospital, 128 08 Prague, Czech Republic; zdenek.kejik@lf1.cuni.cz (Z.K.); Robert.Kaplanek@lf1.cuni.cz (R.K.); Michal.Masarik@lf1.cuni.cz (M.M.); Katerina.Vesela2@lf1.cuni.cz (K.V.); Nikita.Abramenko@lf1.cuni.cz (N.A.); Pavel.Martasek@lf1.cuni.cz (P.M.); 2 BIOCEV, First Faculty of Medicine, Charles University, 252 50 Vestec, Czech Republic; 3 Department of Analytical Chemistry, Faculty of Chemical Engineering, University of Chemistry and Technology, 166 28 Prague, Czech Republic; 4 1st Department of Surgery-Department of Abdominal, Thoracic Surgery and Traumatology, First Faculty of Medicine, Charles University and General University Hospital, 121 08 Prague, Czech Republic; Petr.Dytrych@lf1.cuni.cz (P.D.); David.Hoskovec@lf1.cuni.cz (D.H.); 5 Department of Respiratory Medicine, First Faculty of Medicine, Charles University and Thomayer Hospital, 140 59 Prague, Czech Republic; Martina.Vasakova@lf1.cuni.cz; 6 Institute of Molecular Genetics, Czech Academy of Sciences, 142 20 Prague, Czech Republic; kralova@img.cas.cz

**Keywords:** CTCs, NSCLCs, metastasis suppression, curcumin, flavonoids

## Abstract

Designing optimal (neo)adjuvant therapy is a crucial aspect of the treatment of non-small-cell lung carcinoma (NSCLC). Standard methods of chemotherapy, radiotherapy, and immunotherapy represent effective strategies for treatment. However, in some cases with high metastatic activity and high levels of circulating tumour cells (CTCs), the efficacy of standard treatment methods is insufficient and results in treatment failure and reduced patient survival. CTCs are seen not only as an isolated phenomenon but also a key inherent part of the formation of metastasis and a key factor in cancer death. This review discusses the impact of NSCLC therapy strategies based on a meta-analysis of clinical studies. In addition, possible therapeutic strategies for repression when standard methods fail, such as the administration of low-toxicity natural anticancer agents targeting these phenomena (curcumin and flavonoids), are also discussed. These strategies are presented in the context of key mechanisms of tumour biology with a strong influence on CTC spread and metastasis (mechanisms related to tumour-associated and -infiltrating cells, epithelial–mesenchymal transition, and migration of cancer cells).

## 1. Introduction

Lung cancer is a harmful and dangerous oncological disease responsible for frequent cancer-related deaths [1,2]. Non-small-cell lung cancer (NSCLC) is the most common type of lung cancer, accounting for 85% of those deaths. A significant proportion of NSCLC patients (~40%) have metastatic disease (stage IV) with a poor prognosis (low overall survival (OS) and progression-free survival (PFS)) [3,4,5]. It is well known that the majority of deaths of oncology patients, including those with NSCLC, are not caused by the primary tumour but by metastasis [5]. One of the most potent metastatic factors is circulating tumour cells (CTCs) [6].

The role of CTCs in NSCLC metastasis is briefly described below. Section 2 discusses the influence of CTC count on chemotherapeutic efficiency. Section 3 describes the potential of CTC analysis to improve therapeutic prognosis mainly in terms of programmed cell death protein ligand 1 (PD-L1) expression and epidermal growth factor receptor (EGFR) genotyping. Section 4 is focused on the mechanisms supporting the spread of CTCs, such as tumour-associated and -infiltrating cells, epithelial–mesenchymal transition (EMT), and cellular migration, and discusses the ability of natural agents to suppress them.

CTCs were first observed by Asworth in 1869 [7]. They are released by primary tumours into the bloodstream or lymphatic system and have the potential to form micrometastatic deposits in distant sites [8]. Over time, many patients develop local recurrence or distant metastases. Therefore, the importance of CTCs in NSCLC pathology appears to be very significant. For example, Sienel et al. found that disseminated cancer cells are detectable in approximately 20% of patients with operable NSCLC who have poor clinical outcomes [9]. This finding implies that determining CTC levels can provide useful information about the efficacy of surgery and predict the need for adjuvant therapy. Yoon et al. showed that determining post-surgery CTC levels can help predict the risk of disease progression. CTCs expressing thyroid transcription factor-1 and/or cytokeratin 19 (CK19) were found to be strongly associated with disease progression and PFS [10]. It was shown that 40% of patients (10/25) developed disease progression after surgery when CTCs expressed these markers, and only 4.5% (1/22) had disease progression when these markers were not expressed. Numerous studies have demonstrated that surgical manipulation can promote the dissemination of tumour cells into circulation [11,12,13]. The risk of tumour cell dissemination can also be reduced by suitable operation techniques. Substituting an artery-first group with a vein-first group led to a reduction in incremental CTC change by half [14]. Five-year OS (disease-free survival) was significantly higher for patients who underwent a vein-first operation than for patients who underwent an artery-first operation. In agreement with the strong implications of numerous high-impact clinical studies, a higher CTC level correlated with a higher risk of metastasis and shorter OS [15,16,17]. However, clear cut-offs for such parameters are not yet known.

Due to the different techniques used for CTC isolation and enumeration and the heterogeneous and often small cohorts of patients, guidelines and standards are difficult to establish [18,19]. Examples of published thresholds are shown in Table 1. However, changes in CTC counts during therapy also represent an important marker for modulating cancer treatment.

The CTC count was approximately twice as high for NSCLC patients as for patients with benign lung diseases (pneumonia, pulmonary tuberculosis, bronchiectasis, or pneumothorax) or healthy subjects [20]. The counts for all controls (healthy subjects and patients with other lung diseases) were under the designated limit. In such cases, detection of CTCs is most likely a false positive result due to the extremely large numbers of blood cells in the samples [21,22]. On the other hand, Illie et al. found that CTC-positive patients with tobacco-induced chronic obstructive pulmonary disease may have developed lung cancer [23]. This suggests that cancer patients in the early stages of lung cancer could display high metastatic activity, such as higher CTC counts and formation of CTC clusters (a highly metastatic and aggressive form of CTC) [24,25,26].

Nevertheless, some trials imply that significant CTC levels should be seen only at a late stage of the late/metastatic stages. Chen et al. did not find a significant difference between subjects with benign diseases and healthy subjects [20]. The obtained values were not dependent on age (≤60 years versus >60 years), sex (male versus female), smoking status (former versus current smoker), or pathology type (adenocarcinoma versus squamous cell carcinoma and others). However, the observed CTC counts were significantly lower for patients with stage I or II disease than for those with stage III or IV disease. The difference between patients with stage III and patients with stage IV disease was not significant. In addition, Krebs et al. found that disease progression (from stage IIIa to IV) significantly increased the CTC count associated with metastatic activity [17]. Numerous clinical trials have shown that higher CTC counts correlate with poor therapeutic prognosis (shorter OS and PFS) [16,17,27,28]. Similarly, Wendel et al. observed a correlation between NSCLC development and CTC count [29]. On the other hand, NSCLC patients (early stage) with higher CTC count (≥5/mL) display significantly higher radiotherapy failure and cancer recurrence. [26] However, our knowledge is still limited, and other clinical trials are much needed for better understanding of this phenomenon.

Due to the importance of CTCs in tumour pathology, assessing CTC count can significantly improve cancer prognosis. For example, assessment of CTC count in combination with a panel of plasma tumour markers (carcinoembryonic antigen, neuron-specific enolase, and Cyfra21–1) led to a significant increase in the diagnostic efficacy of the panel [20]. Clinically valuable information can be obtained by CTC-based genotyping. This robust approach can analyse numerous cancer biomarkers. This strategy effectively enables the determination of tumour properties, mainly metastatic activity and drug resistance, and significantly increases the clinical potential of CTC count.

In short, we can say that CTC count and analysis represent revolutionary approaches in diagnostic methods. Whereas the utility of solid biopsy is strongly limited because of tumour heterogeneity, repeated tissue sampling is associated with a significant burden on patients. Liquid biopsies can dynamically and noninvasively interrogate the whole molecular landscape of tumours.

## 2. Influence of CTC Count on Chemotherapeutic Efficacy

Numerous studies have shown a strong association between CTC count and the clinical efficacy of chemotherapy. The CTC count data discussed in this chapter were determined with CellSearch and with 7.5 mL of blood, unless otherwise stated. Zhang et al. found an inverse correlation between the effects of cisplatin doublet therapy and CTC count for NSCLC patients (stage IIIB or IV) [28]. A CTC count of 8 (in 3.2 mL of peripheral blood) or more (15.2% patients) was clinically manifested by decreased PFS (7.4 vs. 5.3 months) and OS (23.1 vs. 9.0 months).

Similarly, Krebs et al. observed that patients (stage IIIA to IV) with CTC counts lower than 5 had more than double the PFS and OS of patients with higher counts [17]. Nevertheless, a change in the number during chemotherapy can have significantly higher prognostic importance than a single count. Patients with decreased CTC counts sometimes had higher OS and PFS than patients with unchanged CTC counts.

In summary, the CTC level is a strong biomarker for the prediction of NSCLC chemotherapy response. However, some works question its clinical application. For example, Zhang et al. did not find any correlation between CTC count and tumour size [28]. Similarly, Hirose did not observe any correlation between the presence of CTCs (one or more) and the number of metastatic sites, tumour burden, or serum levels of lactate dehydrogenase or albumin in NSCLC patients (metastatic stage IV) [30]. The correlation between response to chemotherapy (gemcitabine and carboplatin) and CTC count was found to be statistically insignificant. On the other hand, disease progression was significantly higher in CTC-positive patients (66.7%) than in CTC-negative patients (23.8%). The proportion of patients with stable disease and partial response was significantly higher in the CTC-negative group.

Juan et al. reported that 24% of patients with advanced NSCLC (stage IIIB with pleural effusion or stage IV) before the third cycle of chemotherapy displayed higher CTC counts (two or more) [31]. However, this pattern was not associated with significantly lower OS but with an insignificant improvement in OS. Nevertheless, a reduction in CTCs during chemotherapy led to a better prognosis. Findings regarding the influence of CTCs on chemotherapy are shown in Table 1.

The above implies that baseline CTCs can be used as markers for chemotherapy response; however, determination of CTC count during and after chemotherapy can lead to a better prognosis. Nevertheless, two points limit the utility of CTC count. CTCs, even obtained from a single patient at the same time, may show strong heterogeneity (see Section 3) and thereby varied metastatic potential. In addition, CTCs can exist in clusters (very strong metastatic factors) that can significantly influence cancer development [24].

**Table 1:** Influence of circulating tumour cell (CTC) count on non-small-cell lung cancer (NSCLC) chemotherapy.

Patient Characteristics	Clinical Finding	Ref.
101 patients with stage IIIA, IIIB, or IV disease; platinum doublet chemotherapy (United Kingdom)	CTC count ^1^ for prediction ^2^ (baseline): ≥5; PFS (6.8 vs. 2.4) and OS (8.1 vs. 4.3)	[17]
	CTC count for prediction (after therapy): ≥5; PFS (7.6 vs. 2.4) and OS (8.8 vs. 4.3)	
21 patients with stage IV disease; previous chemotherapy with belagenpumatucel-L, 16 months (USA)	CTC count for prediction: ≥2; OS (20 vs. 5)	[27]
37 patients with stage IIIB disease with pleural effusion or stage IV disease with bidimensionally measurable lesions in a previously irradiated field; docetaxel plus gemcitabine, 28 days ^4^ (Spain)	CTC distribution: ≥1 (58%) ^3^, ≥2 (32%), and ≥5 (8%)	[31]
	CTC count for prediction (baseline): ≥2 (nonsignificant); OS (8.1 vs. 12.2) and PFS (9.4 vs. 4.3)	
	CTC count for prediction (after therapy): ≤ 1; OS (10.1 vs. X ^5^)	
46 patients with stage IIIB or IV disesease; platinum doublet therapy (China)	CTC distribution: ≥1 (87), ≥3 (63), ≥5 (37) and ≥8 (15)	[28]
	CTC count for prediction (baseline): ≥8; (OS (21.3 vs. 9.0) and PFS (7.4 vs. 5.3))	

^1^ The CTC counts discussed in this table were determined with CellSearch and with 7.5 mL of blood. ^2^ Overall survival (OS) and progression-free survival (PFS) shown in months. ^3^ Proportion of patients with a given number of CTCs. ^4^ Length of chemotherapy. ^5^ Study time too short for determination.

Nevertheless, observing the CTC level during and after therapy can provide valuable information about the patient’s response to therapy. As CTCs are an inherent marker of metastatic activity, their increase during therapy strongly indicates a high risk of metastases [32]. In the case of an increasing number of CTCs, alternative chemotherapy may be used; nevertheless, the efficiency of this strategy is limited [33].

It is well known that chemotherapy or radiotherapy failure is associated with not only drug efficiency but also the induction of new aggressive and metastatic forms of oncological diseases [34,35,36]. For example, Shah et al. found that cisplatin application induced EMT (one of the key steps in the mechanism of CTC spreading) via induction of endoplasmic reticulum (ER) stress [34]. The authors observed that following removal of stress, some characteristics of EMT, such as increased vimentin expression, persisted, indicating that the ER stress that induced these phenomena is a long-term effect. Wang et al. found that celecoxib (a selective inhibitor of cyclooxygenase 2 (COX-2)) induces EMT of NSCLC cells via upregulation of MEK-ERK signalling. [37] This fact could explain the failure of celecoxib in clinical trials [38,39,40]. The RAS–Raf–MEK (mitogen-activated protein kinase (MAPK))–extracellular signal-regulated kinase (ERK) pathway is a key signalling pathway that regulates a wide variety of cellular processes, including proliferation, differentiation, apoptosis, and stress responses. MAPKs and ERKs play a crucial role in the survival and development of tumour cells [41,42]. For example, resistance to third-generation EGFR tyrosine kinase inhibitors can be caused by activation of MEK/ERK signalling [43].

Dea et al. reported that doxorubicin treatment can support lung metastasis via suppression of MSDS exosomes in a mouse model [44]. Bhattacharya et al. found that paclitaxel-treated macrophages support angiogenesis and display a higher proportion of M2 macrophages [45].

The abovementioned studies strongly imply that mechanisms responsible for chemoresistance are at least partially responsible for CTC spreading. For example, Atjanasuppat et al. reported that nonadherent H460 cells (an NSCLC cell line) have significantly higher paclitaxel sensitivity than the original line. Both of these phenomena were caused by upregulation of ERK signalling [46]. Lee et al. reported that the hypoxia-related phenotype of A549 cells (an NSCLC cell line) and the hypoxic microenvironment in cancer tissue from NSCLC patients were associated with cisplatin resistance [47]. Similarly, in a breast cancer model, intratumour hypoxia led to the formation of CTC clusters with high metastatic ability [48].

As such, a new therapeutic strategy based on the application of antimetastatic compounds such as migrastatics has been considered [49,50]. These agents are not designed to kill cancer cells, as cytostatics are, but to block cell migration and thus metastatic spread. However, many developed and studied anticancer agents display antimetastatic effects. For example, the Food and Drug Administration (FDA) approved some compounds designed to delay metastatic prostate cancer [51]. In NSCLC, tyrosine kinase inhibitor, immune checkpoint inhibitors, and antibodies against interleukin 6 receptor (IL-6R) have shown antimetastatic effects [52,53,54]. Nevertheless, NSCLC is associated with high heterogeneity; therefore, we recommend the application of multifunctional agents. A higher CTC count or its increase during therapy may be a suitable predictive biomarker for the incorporation of such agents into therapy. Curcuminoids and flavonoids are agents with low toxicity that can target CTCs by various independent mechanisms (see Section 4), and they are potent antimetastatic adjuvant agents [55]. In the future, strategies employing such compounds could lead to earlier adjustment of therapy and increase treatment efficacy.

## 3. CTC Analysis in the Determination of Therapy Prognosis

CTC analysis has potential for the determination and dynamic observation of changes in tumour properties in oncology patients from various perspectives (genomic, transcriptomic, proteomic, and metabolomic) [56]. Such analysis seems to provide critical information for predicting therapeutic response and designing/redesigning optimal therapies for patients.

CTC detection can be based on physical CTC properties (e.g., size, density, and electromechanical characteristics) or tumour-specific epitopes, or CTCs can be detected by high-throughput imaging of unpurified blood cell preparations [57]. CTC analyses are mostly conducted using the FDA-approved EpCAM kit (positive and negative sorting based on epithelial cell adhesion molecule (EpCAM) and the protein tyrosine phosphatase (CD45) receptor, respectively) [32]. However, some obstacles (extremely low CTC numbers and CTC heterogeneity) can limit the promising potential of this approach. CTCs are strongly outnumbered by normal blood cells by a billion-fold, and in obtained clinical samples, there are only a few CTCs.

In addition, CTCs are heterogeneous, and their analysis can provide valuable information for diagnosis [58]. Brung et al. reported that CTC lines (UWG01CTC and UWG02CTC) from patients with gastroesophageal cancer demonstrated rapid tumourigenic growth in immunodeficient mice, and their genotypic and phenotypic profiles were consistent with those of the original tumours [59]. Nevertheless, UWG02CTC cells (EpCAM^+^, cytokeratin^+^, CD44^+^) were much more sensitive to carboplatin, paclitaxel, 5-fluorouracil, doxorubicin, and epoxide than were UWG01CTC cells (EpCAM^-^, low cytokeratin). CTCs are unlikely to be representative of all cancer cells; rather, they correspond to cells with more aggressive metastatic phenotypes [60,61]. On the other hand, CTCs can display high heterogeneity, and their potential for metastasis formation may be very different [62]. Analysis of other CTC phenotypes or the expression and DNA mutation profiles of CTCs may lead to more robust therapeutic prognosis prediction and determination of appropriate therapy.

For example, CTCs expressing programmed death-ligand 1 (PD-L1) constitute a promising biomarker for the design and management of immune checkpoint inhibitor (ICI) therapy. High PD-L1 expression is usually related to a higher histological grade, metastatic activity, and poor prognosis [63]. Satelli et al. reported that PD-L1 expression in the nucleus of CTCs but not the CTC count itself was associated with shorter OS in colorectal and prostate cancer patients [64]. Moreover, the number of CTCs was positively correlated with PD-L1 and cell surface vimentin expression in gastric cancer patients, and higher values were significantly associated with a shorter survival duration and poorer therapeutic response [65]. NSCLC CTCs can display higher levels of PD-L1 than original tumour tissue. For example, He et al. reported that 27% of biopsy samples obtained from lung cancer patients (stage I or II) displayed high expression of PD-L1, while this expression profile was found in the 40% of CTC samples [66]. Dong et al. reported a positive correlation between PD-L1 expression in pulmonary venous CTCs and that in biopsy samples obtained from NSCLC patients (stages I−IV) [67]. However, Janing et al. found strong differences between results from biopsies and CTCs [68]. This finding was most likely caused by tumour heterogeneity and sampling of multiple tumour sites. Most patients in the study had metastatic disease (96%, *n* = 122). Nevertheless, a correlation between higher CTC counts and poor prognosis was confirmed.

Another strategy is to analyse EGFR gene mutations. Hanssen et al. found that NSCLC patients with an altered EGFR genotype displayed higher CTC counts and metastatic activity than patients with EGFR wild-type tumours [69]. Lindsay et al. also observed that NSCLC patients with NG_007726.3 EGFR mutation had higher CTC counts, including a vimentin^+^ CTC (a marker of EMT) phenotype [70]. For example, the proportion of samples with a low CTC count (> 2) with EGFR mutation was only 15%, but 34% of patients with a high CTC count (2 or more) had EGFR mutation. However, this finding is not consistent with studies of tumour samples obtained by biopsy. Nevertheless, the correlations between the mutation and expression profiles in tumours and those in CTCs may increase as the CTC count increases [32].

Importantly, Jiang et al. found high agreement (approximately 90%) between whole-genome sequencing data from DNA samples from primary biopsies and CTCs for oncology patients with pancreatic cancer [71]. Similarly, Heitzer et al. found that patients with stage IV colorectal carcinoma displayed good agreement in the mutation profile (e.g., APC, KRAS, or PIK3CA mutation) [61]. Mutations found in the primary tumour and metastatic tumour were also detected in the corresponding CTCs. However, some mutations were observed only in the DNA obtained from CTCs (hereinafter referred to as CTC DNA). More detailed analysis revealed that these mutations were mostly present at the subclonal level in primary tumours and metastases from the same patients. Similarly, the multiple myeloma frequency of TP53 R273C, BRAF G469A, and NRAS G13D mutations was higher in CTCs than in single cells isolated from tumours with the same aberrant malignant phenotype (CD138^+^ and CD45^−^) with the same procedure [72].

Yanagita et al. published that higher CTC counts were observed in NSCLC patients with detectable oncogenic mutations in either EGFR (*p* = 0.062) or KRAS (*p* = 0.065) than in patients without mutations in these genes in either archival tissue or cell-free DNA [73]. Similarly, oncology patients with a higher number of CTCs were found to have marked changes in ESR1 (*p* <  0.005) and GATA3 (*p* <  0.05) [74].

Relevant clinical information can also be obtained by analysis of the methylation of tumour suppressor genes in CTC DNA or CTC cluster formation. Chimonidou et al. published that the BRMS1 promoter was methylated in CTC samples obtained from oncology patients [75]. This phenomenon was associated with shortened OS and PFS. Yang et al. found that NSCLC patients with a smoking history were more likely to have methylation of BRMS1 [76]. In contrast, patients with high levels of BRMS1 RNA (with a nonmethylated gene promoter) displayed significantly better therapeutic prognoses. Similarly, Schneck et al. observed heterogeneity in the mutation of phosphoinositide-3-kinase (PI3K; e.g., exon 9/E545K or exon 20/H1047R) in CTC DNA obtained from patients with metastatic breast cancer [77]. Because these mutations can significantly decrease the effectiveness of therapy targeting HER2 (EGFR), their analysis provides important insights for the improvement of anticancer therapy.

An important marker strongly associated with the metastatic activity of CTC clusters is plakoglobin (a component of cell junctions). Aceto et al. reported that CTC clusters obtained from oncology patients displayed plakoglobin overexpression (more than 200-fold) compared to single CTCs [78]. CTC clusters are derived from multicellular groupings of primary tumour cells held together through plakoglobin-dependent intracellular adhesion, and although rare compared to CTCs, these clusters greatly contribute to the metastatic spread of CTCs. Patients with at least one and at least three detected CTC clusters were found to have shorter OS and PFS, respectively. Similarly, knockdown of plakoglobin expression in a mouse model suppressed CTC cluster formation and reduced metastatic spread.

Some studies imply that analysing only one factor may not be relevant for determining therapeutic prognosis. One possible solution could be to identify more biomarkers. For example, Sher et al. designed and tested a gene panel (KRT19, ubiquitin thiolesterase, highly similar to HSFIB1 for the assessment of human fibronectin and TRIM28 mRNA) in Taiwanese NSCLC patients (stage IIIB or IV with operable cancer) [79]. The detection rate of this panel was higher (72%) than that of individual genes (41%, 11%, 39%, and 11%, respectively). The detection rate for the CK19 marker was 41%. Patients with higher scores had poor therapeutic responses and worse prognosis (e.g., shorter OS). Other suitable gene panels can be designed based on clinical data obtained from patients bearing other oncological diseases. In patients with breast cancer, co-expression of EpCAM, CD44, CD47, and MET was strongly associated with short OS and a higher number of metastatic sites [80].

The two most frequently studied approaches in NSCLC diagnosis are analysis of PD-L1 expression and EGFR genotyping in CTCs. Dong et al. found that 40.4% and 48.4% of tissue samples from NSCLC patients (stages I-III, after surgery) had EGFR mutations and PD-L1 expression in CTC, respectively [67]. Both of these characteristics are associated with immune checkpoint inhibitor (ICI) and tyrosine kinase inhibitor (TKI) resistance and higher metastatic activity. As such, analysis of CTC PD-L1 and EGFR expression could be used as a predictive strategy for the incorporation of curcuminoids and flavonoids into therapeutic regimens, as these agents target EGFR-related factors, including the T790M mutation and EGFR signalling, via various mechanisms and lower PD-L1 expression and signalling (see Section 4.3).

Both approaches are described and discussed in detail in the next subsections.

### 3.1. PD-L1 in CTCs

Numerous clinical trials have confirmed a strong correlation between PD-L1 expression and the OS and PFS of NSCLC patients [63,68,81,82,83,84,85], which is deeply associated with tumour immunoresistance [86].

For example, the transmembrane protein PD-1 is expressed on immune cells (e.g., B cells, T cells, natural killer (NK) cells, dendritic cells, and regulatory T (Treg) cells) [87]. Interferon gamma (IFN-γ; produced during the immune response) induces overexpression of PD-L1 to protect expressing cells. PD-L1 inhibits inflammatory signalling pathways, suppresses T cells, and prevents autoimmune attack. Nevertheless, tumour tissue protects cancer cells and tumour-associated cells from the immune system. In addition, the expression of these markers in CTCs and CTC clusters is expected to protect them from the immune system and thereby support metastatic spread [88].

On the other hand, anti-PD-1 and -PD-L1 antibodies reactivate the immune system of patients to subsequently eradicate tumours [86].

Tamminga et al. observed a strong influence of CTC level on the efficacy of therapies (mostly nivolumab therapy, 85% of patients) for patients with advanced NSCLC (stage IIIb or IV) [89]. For example, patients with at least one CTC displayed half the durable response rate of patients without CTCs at baseline. In addition, the durable response rate of patients with increased CTC counts was one-sixth that of patients with decreased counts. Similarly, higher CTC levels led to significantly shorter PFS and OS.

In addition, CTC genotyping can increase the utility of PD-L1 expression in NSCLC diagnosis, especially in advanced metastatic tumours. Ilie et al. reported that PD-L1 expression in tumour cells and infiltrating immune cells displayed high agreement with CTC (93%) and white blood cell (73%) counts [90]. Higher PD-L1 expression in strongly correlated with worse OS and PFS. Boffa et al. found that NSCLC patients with higher PD-L1-expressing CTC counts had worse OS (2 years, 31.2% vs. 78.8%, *p* = 0.00159) [91]. According to Dhar et al., the presence of CTCs and the presence of PD-L1-expressing CTCs was negatively correlated with NSCLC patient survival (after treatment with anti-PD-L1 therapy) [92].

Similarly, Janning et al. found that responding NSCLC patients (mostly those with metastatic disease) exhibited either a decrease or no change in their total CTC counts after three or five cycles of therapy (anti-PD-L1 antibody), and primarily resistant patients had an increase in their CTC counts. [68] In contrast, all patients showed an increase in PD-L1-expressing CTCs at progression. A relationship between a higher PD-L1-expressing CTC count (at baseline) and nivolumab resistance for NSCLC patients (mostly after previous chemotherapy) was also reported by Guibert et al. [93].

Nevertheless, a high-impact clinical study published by Nicolazzo et al. revealed a more complicated relationship between PD-L1-expressing CTC count and the prediction of therapy response. PD-L1-expressing CTC counts were determined at baseline and at 3 and 6 months after starting therapy and correlated with outcome [84]. At baseline, 83% of patients (those with metastatic NSCLC previously treated with therapy, most of whom were smokers) displayed PD-L1-expressing CTCs. Overall, 70% of patients with PD-L1-expressing CTCs experienced disease progression or death, while 25% had stable disease or a partial response at the second follow-up time (6 months after starting nivolumab treatment). After three months of treatment, every CTC-positive patient had PD-L1-expressing CTCs. However, no significant difference in therapy prognosis between patients with low and high frequencies of PD-L1-expressing CTCs was found. Conversely, although CTCs were found in all patients after 6 months of treatment, patients could be dichotomised into two groups based on PD-L1 expression in CTCs. Only half of the patients had CTCs that expressed PD-L1, and these patients experienced disease progression, while patients with PD-L1-negative CTCs displayed stable disease or a partial response. This finding implies that 3 months of treatment may not be enough for the activation of the immune system to eliminate PD-L1-expressing CTCs; as such, assessment of PD-L1-expressing CTCs at this time point might not be able to provide relevant therapeutic predictions.

Patients with nonmetastatic NSCLC treated with radiotherapy and chemoradiotherapy showed a small influence of baseline CTC count on therapy prognosis indicators, such as PFS [94]. No significant difference in PFS was found between patients with a high number of CTCs (≥14 CTCs/mL; median PFS 7.4 months) and those with a low number of CTCs (median PFS 9.6 months). In the case of PD-L1-expressing CTCs, a stronger correlation between therapeutic prognosis and CTC count was observed. In chemo-naïve stage IV NSCLC patients, a higher CTC count (>5) at baseline predicted shortened PFS [82].

However, Kulasinghe et al. did not find any correlation between PD-L1-expressing CTC count and disease development in NSCLC patients [95]. This finding implies that PD-L1 expression may not always be a decisive factor in tumour development and metastatic activity. Manjuth et al. found that assessing other factors, such as the expression of mesenchymal markers (e.g., vimentin and N-cadherin), in PD-L1-expressing CTCs can lead to significantly better predictions [96]. Similarly, Schehr et al. found that neutrophils (insignificantly expressing CD45 and expressing PD-L1) can interfere with the assessment of PD-L1-expressing CTCs. The number of CD11b+ cells (CD11b is a neutrophil marker) misidentified as CTCs varied among patients, accounting for 33–100% of traditionally identified CTCs [97]. Another limitation of classical determination of PD-L1-expressing CTC count was shown by Zhang et al. [98]. The authors observed that detection of PD-L1-expressing CTC was not associated with significantly worse prognosis (e.g., PFS). Nevertheless, during nivolumab treatment, the PD-L1 levels sometimes decreased in patients with advanced NSCLC. On the other hand, an increase in the number of therapeutic cycles (four or more) could lead to the detection of aneuploid endothelial CTCs with PD-L1 expression. These cells were associated with disease progression and shorter FPS (5 versus 8 months).

### 3.2. EGFR Genotyping

EGFR (part of the ErbB family) has tyrosine kinase activity [99]. After ligand binding via autophosphorylation, it actively stimulates cellular growth and proliferation. Mutations in its gene can lead to ligand-independent activation, a common occurrence in NSCLC that correlates with poor prognosis. In addition, mutations in lung tumour tissues have also been identified in the CTCs. Marchetti et al. used ultradeep next-generation sequencing to find that 84% of NSCLC patients (stage IIIB or IV) harbouring EGFR mutations in primary tumour tissue also displayed these mutations in CTCs [100], and 13% of patients displayed multiple EGFR mutations (a possible indicator of CTC heterogeneity). No EGFR mutations were observed in the control group.

These mutations can be targeted by TKIs. At present, TKIs represent a promising tool for NSCLC treatment. However, their clinical efficiency can be limited by some EGFR chemoresistance mutations, such as T790M [101,102], whose identification in CTCs represents another strategy that can aid therapeutic design and management. Sundaresan et al. reported that the T790M mutation of EGFR in CTCs correlated with TKI resistance [103]. The agreement between CTCs and tumour biopsies was 74%.

For the treatment of patients with TKI resistance, third-generation TKIs (e.g., AZD9291) have been developed [101]; their efficacy inversely correlates with the CTC level. Yang et al. reported that higher CTC counts predicted poor therapeutic response for NSCLC patients (stage IIIB or IV with the EGFR T790M mutation and TKI treatment failure) [104]. Similarly, the combined effect of erlotinib and pertuzumab was inversely correlated with CTC count, and a decreased CTC count was associated with an approximately two-fold longer PFS [105]. The fact that gene mutations in either EGFR or KRAS have been found to be associated with a higher CTC count could confirm the hypothesis that CTCs represent a more dangerous (aggressive or metastatic) subpopulation of cancer cells.

KRAS (a small GTPase and member of the RAS protein family) transmits signals from transmembrane receptors such as EGFR into cells [106]. It participates in many critical cellular processes, such as proliferation, differentiation and survival. Approximately one-third of patients with NSCLC have a mutation in KRAS. Some studies have found an association between KRAS mutations and higher metastatic activity [107,108]. Oncogenic KRAS mutations constitutively activate downstream signalling pathways (e.g., the MAPK/ERK and PI3K pathways) [109,110].

On the other hand, patients with EGFR DelEx19 mutation showed a good response to therapy (strong decreases in CTC counts associated with increased PFS and stable disease). These findings were provided by a study by Breitenbuecher et al., who reported that 40% of patients lost the EGFR DelEx19 mutation in CTCs during therapy [111]. This loss was associated with better therapy prognosis, as it prolonged the median time to therapy failure from 116 to 355 days.

High level of circulating tumour DNA (ctDNA) is a strong marker of bad prognosis [112]. Unlike CTC, very low concentrations of free DNA (5–10 ng/mL) can observed in the plasma of healthy subjects [113]. Nevertheless, during tumour development (from beginning to advanced), its amount is significantly increased, and it is called ctDNA [114]. ctDNA display higher similarity with DNA obtained from the tissue biopsy sample than CTC DNA [73]. CT contains specific mutations identical to those found in the primary tumour and its metastases [115]. Yanagita et al. reported that CTCs displayed no T790M EGFR mutation despite the mutation being present in tissue biopsy and circulating tumour DNA samples from erlotinib-treated patients (with advanced NSCLC) [73]. A correlation between CTC count and PFS was not observed. However, Sundaresan et al. reported that a combination of CTC genotyping and circulating tumour DNA assessment displayed a higher sensitivity than tissue biopsy assessment [103]; 35% of patients with negative or indeterminate biopsy results had positive results with the combination.

These findings suggest that analysing the EGFR gene in CTCs represents a promising method for the design and management of NSCLC therapy, especially in the case of TKI therapy (Table 2). Nevertheless, resistance to TKIs can also be caused by factors other than mutations in the EGFR gene (e.g., mutations in KRAS and lymphoma-like 11 (BIM)) [104,116]. BIM deletion polymorphisms, such as those producing BIM-γ, are associated with TKI resistance in NSCLC patients harbouring EGFR-activating mutations [117]. Isobe et al. reported that the expression of BIM-γ in CTCs (after therapy) was negatively correlated with both the response of NSCLC patients with EGFR mutations (exon 19 deletion or the L858R mutation) to osimertinib therapy and their PFS [116]. A clinical response was achieved in 27% and 73% of patients with high and low BIM-γ expression, respectively, while 60% and 40% of patients with high and low EGFR expression showed a clinical response. Therefore, more clinical trials are needed for better validation and understanding of this phenomenon and optimization of therapeutic regimens.

## 4. Flavonoids and Curcuminoids for Suppressing the Spread of CTCs

**Figure 1:**
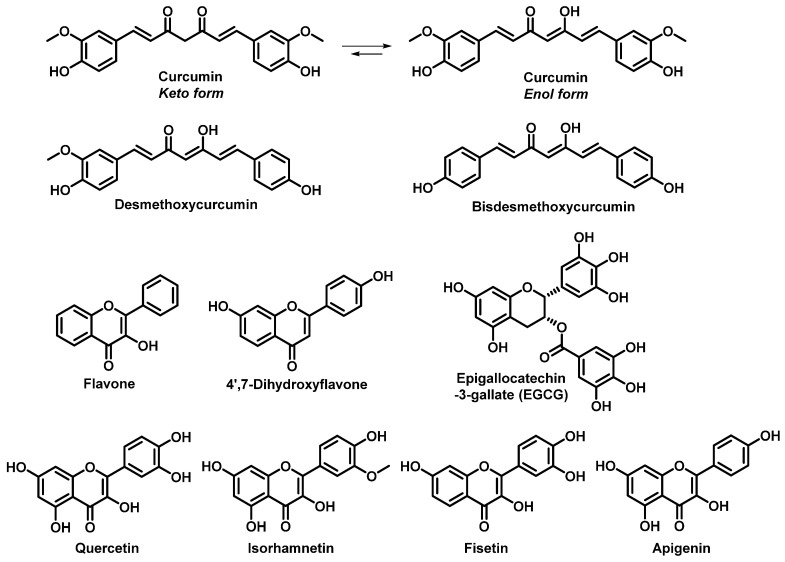
Structure of curcuminoids and flavonoids tested for suppression of NSCLC metastasis.

For most cancer patients, metastases are the leading cause of death. Therefore, migrastatics have been developed, and their administration does not lead to shrinkage of tumours but to suppression of CTC spreading and thus metastatic activity [49]. Some high-impact studies have shown the low toxicity of polyphenols such as curcumin and flavonoids (Figure 1), suggesting that they are very promising agents for this purpose.

Curcumin and other curcuminoids are derived from turmeric (*Curcumin longa*) and have various anticancer effects. Their incorporation in the treatment of lung cancer (chemotherapy and radiotherapy) has led to significant improvement of patient quality of life [119]. Curcumin is one of the most studied agents used for the treatment of various cancers, and antimetastatic effects have also been observed for other natural and synthetic derivatives [120,121,122,123,124,125,126,127,128], implying high potential for incorporation into therapeutic regimens for NSCLC treatment.

Flavonoids are low-toxicity polyphenols that are usually obtained from fruits and vegetables and have great prospects for the treatment of lung cancer. For example, the results published by Sun et al. indicated that daily flavonoid intake could decrease metastatic activity and increase survival in NSCLC patients (stage IIIB or IV) [129]. The use of agents such as epigallocatechin-3-gallate (EGCG) is feasible and safe even at high concentrations, [130] and they have potential for incorporation into radiotherapy regimens. Zhao et al. reported that EGCG can effectively alleviate acute radiation oesophagitis in patients with advanced lung cancer without obvious side effects [131].

In addition, curcuminoids, flavonoids and other agents have the potential to be radiosensitisers, thus increasing the therapeutic efficacy of radiotherapy [132,133,134]. Furthermore, chemotherapy may improve the effectiveness of cytostatics by affecting mechanisms of resistance, tumour cell migration and the stem cell phenotype [123,133,135,136,137].

These results imply, in accordance with many high-impact studies, that curcuminoids and flavonoids have a strong ability to suppress metastasis. Based on their effects on cancer cells and tumour tissues, possible therapeutic strategies implementing curcuminoids and flavonoids can be developed for the repression of NSCLC metastasis, normalization of the tumour microenvironment, repression of EMT, and targeting of migrating cancer cells. The effects of curcumin and flavonoids on these phenomena are described in detail in the following subsections.

### 4.1. Effects of Curcumin and Flavonoids on Tumour-Associated and -Infiltrating Cells: Suppression of CTC Support

The tumour microenvironment contains tumour support/tumour-associated cells, such as macrophages, lymphocytes, fibroblasts, and endothelial cells, and an extracellular matrix with signalling molecules [138,139]. While “healthy” stromal cells can repress carcinogenesis, interactions of cancer cells with the tumour stroma have a strong effect on tumour development, progression, and resistance. In addition, it was observed that CTCs cannot migrate on their own and rather migrate in clusters with tumour-supporting/tumour-associated cells, the metastatic activity of which is an order of magnitude higher than that of CTCs alone [8,24,140]. Therefore, targeting this circulating microenvironment [60] is an intensively studied method of cancer treatment.

The fact that curcumin administration in patients with cancer, including lung cancer, is strongly associated with a decreased level of inflammatory factors (interleukin 6 (IL-6), interleukin 8 (IL-8), and tumour necrosis factor alpha (TNF-α)) implies its strong potential for metastasis suppression [119]. High IL-6 activity is correlated with poor prognosis and lung-cancer-related symptoms such as fatigue, thromboembolism, cachexia, and anaemia [141]. In lung cancer, high IL-6 activity is associated with overactivated signal transducer and activator of transcription 3 (STAT3) signalling (one mechanism of TKI resistance) [142,143], which can lead to IL-6 overproduction and inflammation associated with tumour resistance and development [144]. Tumour inflammation is induced by reciprocal interactions of tumour cells and tumour-associated macrophages (TAMs, the most abundant immune cells in NSCLC), followed by stimulation of TAM polarization to the M2 phenotype and repression of the M1 phenotype [145]. The M1 (antitumour) TAM phenotype is associated with good prognosis, and the M2 phenotype (stimulated by IL-6, IL-8, and other inflammatory factors) is associated with shorter OS [146]. According to a study by Almatroodi et al., the expression of M2 markers (CD68 and CD163) was increased in NSCLC tumour tissue compared to a control (non-tumour tissue from the same patient) [147]. However, expression of M1 markers was decreased in patients with adenocarcinoma and squamous carcinoma; serum levels of interleukin 1 beta (IL-1β), interleukin 4 (IL-4), IL-6, and IL-8 were higher in patients with large-cell carcinoma than in healthy controls.

Some studies imply that the anticancer effect of curcumin may be associated with repression of the M2 TAM phenotype [148,149,150]. For example, a sublethal dose of nanoformulated curcumin (c_max_ 0.61 µmol/l in mouse plasma) or curcumin combined with epicatechin gallate and resveratrol can revert the M2 TAM phenotype to a tumouricidal phenotype with a potent immune antitumour response, leading to tumour eradication [148]. The decreased levels of inflammatory factors (IL-6, IL-8, and TNF-α) strongly associated with curcumin administration (180 mg/day; ~c_max_ 0.5 µmol/l in human plasma [151]) in patients with cancer, including those with lung cancer [119], imply a possible reduction of the M2 TAM phenotype. Higher levels inflammatory factors can increase the M2 TAM phenotype [152,153]. Reduction of their levels in a mouse model of lung cancer led to upregulation of the M1 TAM phenotype [152]. Zou et al. reported that curcumin application to patients with lung cancer leads to a transformation of Treg cells into Th1 cells and an increase in IFN-γ [154]. It is known that the secretion of IFN-γ by Th1 cells leads to macrophage polarization into the M1 phenotype [155]. Nevertheless, the effect of curcumin on the macrophage phenotype in NSCLC patients must be evaluated in other clinical studies.

Myeloid-derived suppressor cells (MDSCs) are cells of the immune system that can play important roles in metastatic spread [156]. Activated MDSCs (e.g., activated by vascular endothelial growth factor (VEGF) or IL-6) induce suppression of innate and adaptive immune systems and thereby the host antitumour response. The blood level of MDSCs is a predictive marker. For example, Augustyn et al. reported that serum levels of cancer-associated macrophage-like cells (CAMLs; multinuclear myeloid cells) can significantly influence the treatment outcome of NSCLC patients (those with advanced cancer treated with chemoradiotherapy and atezolizumab) [157]. The authors found that the levels after the chemoradiotherapy cycle correlated with the metastatic disease status and survival.

Interestingly, curcumin administration in a mouse model of carcinoma led to the maturation of MDSCs (loss of immunosuppressive effects) in spleen and tumour tissues [158]. Additionally, the levels of CD4+ and CD8+ T cells were restored. In MDSCs, this effect was associated with suppression of reactive oxygen species (ROS), arginase (Arg-1), and inducible nitric oxide synthase (iNOS). Lio reported that curcumin could support anticancer immunity by repressing the expression of PD-L1 in cancer cells [159].

An important immunosuppressive effect of MDSCs is the induction of CD4+ T cell differentiation to Treg cells. Via cytokines (e.g., transforming growth factor beta 1 (TGF-β1)), Treg cells suppress cancer-specific effector immune cells (CD8+ T cells) and decrease the antitumour capacity of the host [160]. According to a study by Zou et al., patients with lung cancer have significantly higher Treg cell levels [154]. Curcumin administration at 1.5 g per day significantly decreased Treg cells and increased Th1 cells in the peripheral system. An in vitro study showed that curcumin converted Treg cells obtained from patients into Th1 cells (which induce cancer cell apoptosis) [161] via repression of FOXP3. Experiments in a mouse model with lung metastasis suggested that this strategy could prolong the survival of patients with metastatic disease [162].

Cancer-associated fibroblasts (CAFs) constitute a major portion of the reactive tumour stroma and play a crucial role in tumour progression [163]. They initiate angiogenesis (via overproduction of VEGF), promote tumour progression, and support invasiveness [164]. In addition, some studies suggest that there is an association between CAFs and characteristics of the stem-cell-like phenotypes of NSCLC cells, such as chemoresistance and overproduction of inflammatory factors [165,166,167]. Sung et al. reported that netrin-1 secretion by CAFs leads to overexpression of IL-6 and IL-8 by cancer cells [168]. In a mouse model, administration of the netrin-1 antibody significantly repressed tumour growth. On the other hand, solid tumours after radiotherapy can display increased CAFs [169]. Cho et al. found that the survival of quiescent cancer cells induced via oncogenic signalling factors (e.g., IL-1β, IL-8, TGF-β1, and epidermal growth factor (EGF)) stimulated fibroblast migration to cancer cells and their transformation into CAFs [170]. Some studies imply that CAF metastatic effects could also be associated with CTC migration and survival [135,171,172]. Otero et al. observed CAF−CTC clusters in blood samples from patients with metastatic cancers such as NSCLC [171]. The interaction of such clusters with cancer cells via direct contact or signalling factors can induce drug resistance and cell proliferation and thereby enhance their metastatic potential [135].

Ba et al. reported that the administration of 10 µM curcumin modulated the phenotype of CAFs into one of peritumour fibroblast-like cells via downregulation of the expression of alpha smooth muscle actin (α-SMA), a marker of the CAF phenotype [173]. This transformation led to inhibition of the secretion of procarcinogenic cytokines, including TGF-β1, matrix metalloproteinase 2 (MMP-2), and stromal-cell-derived factor-1 (SDF-1). In accordance with the above findings, Wang et al. showed that curcumin-treated CAFs lost the ability to induce the metastatic potential of cancer cells (a primary cell line derived from patients with oral squamous cell carcinoma) compared to nontreated CAFs [174]. Their ability to interact with cancer cells via gap junctions was also reduced. Another study indicating that curcumin suppresses CAF communication with cancer cells was published by Kreutz et al. [175]. They found that administration of curcumin (30 μM) in coculture with CAFs and cancer cells suppressed TNF-α signalling and survival pathways. However, in single-cell-type cultures, these effects were not observed. Luo et al. reported that CAFs obtained from NSCLC patients (stages I–III) induced EMT of NSCLC cells (A549 and H1299) [176]. This pattern was associated with a metabolic transition of NSCLC cells to aerobic glycolysis in association with the connexin 43 gap junction. Subsequently, overactivation of PI3K/protein kinase B (Akt) and MAPK/ERK signalling and increased mobility and invasiveness of cancer cells were observed. The expression of CAF markers in tumour tissue (α-SMA, lactate dehydrogenase isoform B, and connexin 43) was also strongly correlated (*p* < 0.0001) with poor prognosis, and sometimes shorter OS and PFS.

Tumour endothelial cells (TECs) support nutritional transport to tumour tissue by inducing angiogenesis (via VEGF) and assist in leukocyte infiltration [177]. Higher levels of TECs can lead to chemoresistance and higher metastatic activity. In a mouse NSCLC model, targeting the vascular endothelial growth factor receptor (VEGFR) and EGFR pathways overcame TKI resistance and suppressed angiogenesis [178,179]. Similarly, Lee et al. showed that targeting TECs could repress the paclitaxel resistance of NSCLC brain metastases [180]. Unlike normal cells, TECs display wide and leaky junctions, multiple transendothelial channels, and abnormal shunts, which contribute to the high permeability of the tumour vasculature. This phenotype transition is stimulated by VEGF [181,182].

**Figure 2:**
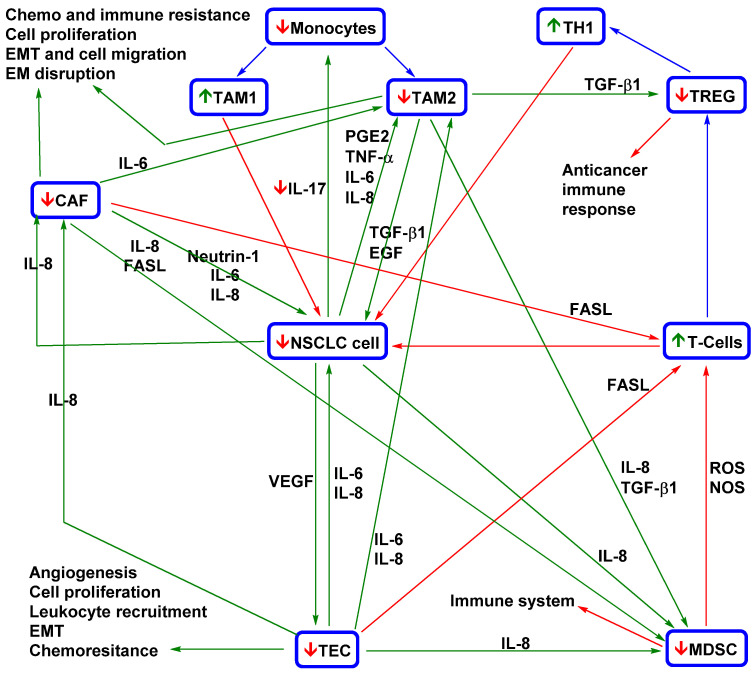
Simplified model of curcumin and flavonoids effects on the NSCLC microenvironment [83,119,148,158,159,162,168,170,174,175,186,187,188,189,190,191,192,193,194,195,196]. A necessary part of igenesis and CTC spreading is the interaction of NSCLC cells with tumour-associated cells. NSCLC cells recruit monocytes via IL-17 into tumour tissue. Signalling factors (e.g., IL-6, IL-8, TNF-α, and PGE2) produced in the tumour microenvironment stimulate monocyte differentiation into TAM2 (which support NSCLC cell proliferation, EMT, chemoresistance and immune resistance, and EM disruption). MDSCs recruited via IL-8 and TGF-β1 repress the cytotoxic effects of T cells against NSCLC cells and induce their differentiation into Treg cells that are responsible for the suppression of the host immune response. VEGF-recruited TECs affect EMT, chemoresistance, and the proliferation of NSCLC cells, recruit MDSCs and induce angiogenesis. IL-8-activated CAFs decrease the anticancer immune response (with the support of TAM2 and MDSCs) and repress T cells. CAFs stimulate EMT and induce NSCLC cell proliferation, migration, and drug resistance. Tumour-associated cells help sustain the tumour microenvironment and aggressive metastatic phenotype. TAM2, CAFs, and TECs are co-inducers of EMT and thereby CTC spreading, chemoresistance, and immune resistance. MDSCs and Treg cells repress the host immune response and thereby support CTC survival in the blood. Nevertheless, the anticancer effect of curcuminoids and flavonoids is not dependent on targeting of NSCLC cells, as they repress other important parts of the complex tumour ecosystem. The application of such agents is associated with stimulation of the immune system (higher levels of TAM1 and T cells; lower levels of Th1 cells, TAM2, and Treg cells; and lower recruitment of monocytes and MDSCs). Curcuminoids and flavonoids also decrease the levels of CAFs and TECs and repress their interaction with NSCLC cells. In addition, the application of curcuminoids and flavonoids leads to a decrease in the proliferation, survival, chemoresistance, immunoresistance, and migration ability of NSCLC cells. CAF, cancer-associated fibroblast; EM, extracellular matrix; EMT, epithelial–mesenchymal transition; EGF, endothelial growth factor; FasL, Fas ligand; IL-1β, interleukin 1β; IL-6, interleukin 6; IL-8, interleukin 8; PGE2, prostaglandin E2; NOS, nitric oxide species; ROS, reactive oxygen species; TAM1, tumour-associated macrophage M1; TAM2, tumour-associated macrophage M2; TEC, tumour endothelial cell; TGF-β1, transforming growth factor beta 1; Th1, T helper 1; Treg, regulatory T; TNF-α, tumour necrosis factor alpha; VEGF, vascular endothelial growth factor. Green arrow = induction/activation of factor/phenomenon/cell; red arrow = repression/inhibition of factor/phenomenon/cell; blue arrow = differentiation of immune cells; **↑** = curcumin/flavonoids activation/induction; **↓** = curcumin/flavonoids repression/inhibition.

High levels of B cell lymphoma 2 (Bcl-2), a key antiapoptotic protein, were shown to promote [181,182] tumour cell proliferation and invasion [181,183]. A study in a mouse model indicated that this ability is not dependent on the tumour mass. Another important metastatic TEC function was reported by Yadav et al. [184]. They found that endothelial cells overexpressing Bcl-2 (EC-Bcl-2) can display a higher affinity for cancer cells via overexpressed E-selectin and can decrease the apoptosis of CTCs. In the mouse model, coadministration of cancer cells with EC-Bcl-2 led to significantly higher metastatic activity. This implies that tumour-associated endothelial cells can enhance the survival of tumour cells in the blood and chaperone them to distant sites. However, their function can be significantly repressed by curcumin, and more effectively repressed by curcumin in combination with flavonoids, such as EGCG [185]. Such applications can lead to repression of angiogenesis via decreased VEGF production [186,187,188], blocking monocyte binding via downregulation of nuclear factor kappa-light-chain-enhancer of activated B cells (NF-kB) signalling [189]. A model of the therapeutic effects of curcumin and flavonoids on the tumour microenvironment and tumour development is shown in Figure 2.

CTC spreading from tumours is not an isolated phenomenon but a central part of the complex process underlying the development of metastases. Tumour-associated cells can support CTC metastatic activity in several ways. As part of the tumour microenvironment, they can induce an aggressive metastatic phenotype with high production of CTCs (e.g., an EMT phenotype), protect CTCs in the bloodstream, and assist in metastasis formation (see the next subchapter). These phenomena suggest that repressing tumour-associated cells could be an important part of CTC targeting. For example, higher lymphocyte infiltration in breast cancer patients was associated with higher CTC counts and metastatic relapse [197]. Additionally, higher Treg cell levels and neutrophil-to-lymphocyte ratios can induce CTC spreading [198,199]. CTCs in clusters with CAFs display higher survival in the bloodstream [135]. Osmundski et al. found that TAM-associated macrophages can stimulate an aggressive phenotype of prostrate CTCs, including high adherence and plasticity [200]. In breast cancer, a decrease in CD8+ T cells and IFN-γ can lead to an increase in the CTC count [201]. On the other hand, activated NK cells can repress metastasis via CTC killing [202].

In addition, the application of curcuminoids and flavonoids can significantly lower CTC counts [203,204]. Because these agents are multifunctional, their effects on tumour-associated cells should also be considered; these are shown in Table 3.

The above results show that curcuminoids and flavonoids repress CTC spreading induced by the tumour microenvironment and metastatic activity by targeting tumour-associated cells, including circulating cells. Relevant in vivo clinical trials show that the application of curcuminoids and flavonoids could greatly enhance NSCLC treatment [119,131,219]. However, the above data were mostly obtained from animal models of various oncological diseases. Therefore, more clinical trials are required to design therapeutic applications.

### 4.2. Effect of Curcumin and Flavonoid Applications on EMT and Metastasis Formation

Two important phenomena that are strongly associated with CTC spreading in lung cancer are EMT and mesenchymal–epithelial transition. First, EMT (induction of the TAM M2 phenotype, CAFs, and TECs) causes polarity loss and cell/matrix adhesion of cancer cells, aids digestion of the extracellular matrix, and supports migratory properties (e.g., actin polymerization) [220]. In the next step, cancer cells are taken up in the bloodstream and become CTCs. CTCs are expected to undergo transitions. In the metastatic site or in the bloodstream, CTCs undergo mesenchymal−epithelial reverting transition (MErT) and obtain increased cell adhesion, and the most aggressive of them can form metastatic tumours [221]. CTC spreading can also be stimulated by macrophages, which provide immunoprotection and growth promotion for the formation of metastases [144]. Manjunath et al. found that a higher number of CTCs with expression of PD-L1 and mesenchymal markers (vimentin and N-cadherin) was significantly associated with reduction of PFS [96]. Another important role of EMT in metastatic spread could be stimulation of resistance of CTCs to treatment. For example, PD-L1-expressing CTCs obtained from NSCLC patients displayed spindle-like elongated morphology, which corresponded to EMT-associated nivolumab resistance [84,222]. PD-L1-negative CTCs were mostly small and regularly shaped (round). Raimondi et al. reported the coexpression of vimentin with PD-L1 in these cells [222].

Relevant studies imply that curcumin and flavonoids can repress the mesenchymal phenotype. Liang et al. reported that curcumin administration (100 mg/kg) reversed tobacco-smoke-induced EMT alterations in a mouse model [223]. This process was associated with increased expression of epithelial markers (E-cadherin) and decreased expression of mesenchymal markers (vimentin and N-cadherin). Similarly, Chang et al. found that quercetin repression of EMT via Akt downregulation was strongly associated with a reduction in bone metastasis in an NSCLC mouse model [224].

Knowledge about MErT is still limited. Several studies suggest that E-cadherin overexpression, which enables CTCs to adhere to target tissue and survive within ectopic metastatic microenvironments, is key in the MErT process [221,225,226,227]. MErT can also be significantly influenced by the presence of TAMs. Yang et al. reported that the M1 TAM phenotype can support MErT by stimulating E-cadherin expression and thereby enhance cancer cell colonization [228]. Infiltrating cancer cells that penetrate healthy tissues can likely survive in the dormant state even with a lower metabolic load. Their eradication is complicated.

Survival of dormant cancer cells can occur through induction of the hypoxia phenotype and NRF2 and their activation by Wnt/β-catenin signalling [229,230]. In the case of micrometastasis, cancer cell development and survival are strongly dependent on higher ROS levels [231]. Curcuminoids and flavonoids can repress oxidative stress and inflammatory factors in healthy tissues and cells [232,233,234,235] and are potent inhibitors of Wnt/β-catenin signalling [236,237]. For example, Tabasum et al. reported that fisetin (10 µmol/l) inhibited the expression of β-catenin, NF-κB, EGFR, and STAT3 and decreased stem cell phenotype markers (CD44 and CD133) and TKI resistance in NSCLC cell lines (A549 and H1299) [236]. Targeting STAT3 was found to block TEC activation by cancer cells in metastatic brain sites and thereby suppress metastasis formation [238]. In addition, curcumin and flavonoid administration (apigenin, flavone, and 4′,7-dihydroxyflavone) can modulate the hypoxic phenotype of NSCLC cells [239,240].

**Figure 3:**
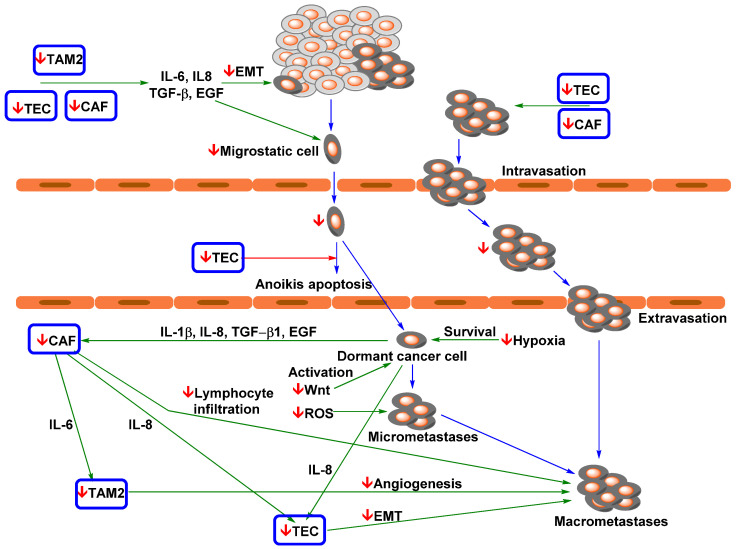
Simplified model of curcumin and flavonoids action on NSCLC metastasis [83,190,223,224,229,230,231,236,237,238,239,240,242,243,244,245,246,247,248,249]. 1. NSCLC cells with a mesenchymal phenotype partially induced by TAM2, TECs, and CAFs migrate from tissue into blood vessels. 2. CTCs are transported (via passive transport) to distant metastatic site(s), and TECs can serve as guardians against anoikis apoptosis. 3. Infiltrating NSCLC cells revert to an epithelial phenotype via MErT (which possibly occurs in blood). 4. Dormant cancer cells activated by Wnt signalling start recruiting CAFs and TECs and form micrometastases. 5 Micrometastases stabilised by oxidative stress recruit tumour-associated cells to form macrometastases, at which point metastatic NSCLC can be diagnosed in patients. TECs and CAFs can support the formation of highly aggressive and metastatic CTC clusters. CAF, cancer-associated fibroblast; EMT, epithelial–mesenchymal transition; EGF, endothelial growth factor; IL-1β, interleukin 1β; IL-6, interleukin 6; IL-8, interleukin 8; PGE2, prostaglandin E2; ROS, reactive oxygen species; TAM2, tumour-associated macrophage M2 phenotype; TEC, tumour endothelial cell; TGF-β1, transforming growth factor beta 1; VEGF, vascular endothelial growth factor. Green arrow = induction/activation of factor/phenomenon; red arrow = repression/inhibition of factor/phenomenon; blue arrow = transition between individual steps of metastatic spread; **↓** = curcumin/flavonoids repression/inhibition.

Importantly, the iron chelation ability of flavonoids may play a significant role in repressing micrometastasis. Fryknäs et al. reported that iron chelators are potent agents for targeting quiescent cancer cells [241]. The suppressive effects of curcumin and flavonoids on metastatic spread and development are shown in Figure 3.

In this process, in addition to targeting signalling pathways of NSCLC cells, curcumin can modulate processes in tumour-associated cells. The presence of TAM2 in the tumour microenvironment of lung adenocarcinoma contributes to an invasive phenotype and induces the expression of EMT-related markers [250]. Zhang et al. reported that curcuminoids are able to reverse the macrophage phenotype from TAM2 to TAM1 by inhibiting STAT3 signalling [251]. In glioblastoma, curcumin treatment elicited activation of antitumour STAT1 signalling and the expression of iNOS in TAMs [149]. Mukherjee et al. found that a sublethal dose of TriCurin (curcumin in combination with resveratrol and epicatechin gallate; ~ nM concentration in plasma) in a mouse model changed TAMs to the M1 phenotype and was associated with strong repression of tumour growth [150]. Nevertheless, Murakami et al. found that curcumin inhibited lipopolysaccharide-induced expression of inducible forms of both nitric oxide synthase and cyclooxygenase in RAW 264.7 cells (murine macrophages) [252]. This finding could mean that curcumin could also target micrometastases by lowering inflammatory processes and oxidative stress.

Zeng et al. found that curcumin induces endoplasmic reticulum stress, loss of mitochondrial potential, cell cycle arrest at the G2−M transition, and apoptosis in prostate CAFs. The sensitivity of natural fibroblasts was approximately three times lower than that in treated CAFs [253]. Ba et al. reported that a sublethal curcumin dose (10 µM) decreased the expression of α-SMA, MMP-2, SDF-1, and TGF-β1, and Smad2/3 phosphorylation/activation by approximately half [173]. Curcumin can also suppress CAF-induced EMT of cancer cells. Du et al. found that in CAFs, monoamine oxidase A (a mitochondrial enzyme) induces mammalian target of rapamycin (mTOR)/hypoxia-inducible factor 1 alpha (HIF-1α) signalling [217]. In prostate cancer cells, this CAF phenotype can induce ROS production and C-X-C motif chemokine receptor 4 (CXCR4) and IL-6 receptor expression and thereby cell migration and invasion. Nevertheless, curcumin application suppresses the activation of HIF-1α signalling and subsequently suppresses EMT in cancer cells. In a mouse model of pancreatic cancer, it was observed that curcumin-induced EMT suppression via CAF targeting could repress cancer metastasis [174].

Recruitment of TECs by cancer cells is associated with activation of JAK/STAT3 signalling via IL-8. Nevertheless, application of curcumin in combination with EGCG (50 mg/kg per day) in a mouse model of colorectal carcinoma led to suppression of JAK/STAT3 signalling in endothelial cells and thereby their recruitment by cancer cells [185]. Similarly, the therapeutic effect of curcumin in glioblastoma also causes a reduction in TEC recruitment [254]. TEC presence was found to correlate with EMT and metastasis in various cancer models [255,256,257]. These effects, however, may be due to recruitment of leukocytes by TECs. NSCLC patients can have significantly increased blood levels of TNF-α [258]. Kumar et al. reported that endothelial cells induced TNF-α expression via activation of products of NF-KB signalling (adhesion factors; e.g., intracellular adhesion molecule-1 (ICAM-1), vascular cell adhesion molecule-1, and endothelial leukocyte adhesion molecule-1) [189]. Nevertheless, curcumin can effectively revert this phenotype of endothelial cells and subsequently monocyte adhesion.

In addition, some studies suggest that curcumin also repressed cluster formation. Taftaf et al. reported that depletion of ICAM-1 in a mouse patient-derived xenograft (PDX) model of triple-negative breast cancer significantly inhibited cluster formation, tumour cell transendothelial migration, and lung metastasis [259]. Yang et al. found that curcumin inhibited the IL-6/STAT3 pathway in NCI-H446 and NCI-1688 small carcinoma cells, leading to suppression of VEGF, MMP-2, MMP-7, and ICAM-1 [260]. Nevertheless, in A549 cells, IL-1β can induce ICAM-1 expression via the NF-kB and Src/PDGFR/PI3K/Akt pathways [261,262]. However, in the presence of curcumin, ICAM-1 expression is repressed [262].

One of the key factors that control CTC acceptance and possible micrometastasis formation is induction and stimulation of the supportive microenvironment (called pre-metastatic niche [263]) in distance organs by primary tumour [264]. Tumour cells display an ability to selectively modify the microenvironment of distant organs via extracellular vehicles (e.g., exomes). They are nano-sized membranous structures liberated from cells into extracellular space [265]. Exosomes transport proteins, RNA, DNA, miRNA, and lipids. Exosomes can mediate communication between different cell types from various tissue and organs.

Numerous high-impact studies imply that tumour exosomes strongly support tumourgenesis and metastatic formation [264]. Ma et al. reported that exosomes isolated from NSCLC patients displayed significantly higher levels of some miRNAs (e.g., miR-3157-3p, miR-3613-5p, and miR-3921) against healthy controls [266]. In addition, metastatic patients have higher expression of miR-3157-3p (exosomes and tumour tissues) than comparative nonmetastatic ones. A549 cells with transferred miR-3157-3p display an increase in protein levels of VEGF, MMP2, and MMP9, and their exosomes can induce vascular permeability of endothelial cells and angiogenesis. In the mice model, exosomes with miR-3157-3p stimulate higher microvessel density and larger tumour tissue.

It was proven that curcuminoids and flavonoids are potent suppressor of MMPs actives and VEGF signalling [267,268]. This suggests that they could suppress the role of exosomes in the formation of the metastases. Kaplan et al. found that bone-marrow-derived haematopoietic progenitor cells (BMDCs) with expression of vascular endothelial growth factor receptor 1 (VEGFR1) significantly participate in the formation of pre-metastatic sites in mice with Lewis lung carcinoma [263]. VEGFR1 repression by specific antibody suppresses BMDCs clusterisation and prevents tumour metastasis. Their cluster formation in the pre-metastatic site was associated with MMP9 induction. Nevertheless, their effect on tumour-derived exosomes was much more complicated. It was observed that curcumin-exposed cancer cells liberated exosomes. It has been shown that treatment of cancer cells with different doses of curcumin leads to the release of exosomes containing curcumin [269].

Unlike classical tumour exomes, curcumin-induced exosomes display anticancer effects in recipient cells and reduce tumour growth. For example, exosomes produced by H1299 cells (10 μM curcumin for 48 h) display upregulation of the transcription factor 21 (TCF21) via downregulation of DNA Methyltransferase 1 [270]. The level of the TCF21 mRNA is inversely correlated with poor prognosis in patients with lung adenocarcinoma [271] and the aggressivity of NSCLC cell lines [270]. In the presence of exosomes derived from curcumin-pre-treated H1299 cells, BEAS-2B cells displayed a significant decrease in proliferation, colony formation, and migration.

Exosomes derived from K562 leukemic cells increase production of IL8 and VCAM1 in endothelial cells; nevertheless, after curcumin treatment (20 μM for 24 h), the obtained exosomes displayed opposite effects and inhibited tube formation and vascular permeability in the endothelial cells [272]. In K562 cells, curcumin caused a decrease of cellular miR-21, while it increased miR-21 selective packaging in exosomes. Its decrease can induce PTEN expression (target of miRNA-21) [273]. In the endothelial cells, higher levels of miR-21 can supress their angiogenic capacity, directly targeting RhoB, a critical regulator of actin dynamics [274].

Exosomes derived from the TS/A cell line (murine mammary adenocarcinoma) displayed a strong inhibition effect on cytotoxicity of the IL-2 activated NK cells [275]. Nevertheless, exosomes isolated from the cells pre-treated with polyphenols (curcumin, baicalin, or genistein, 1 µM for 36 h) cause a significant reduction of immunosuppression of NK cells and increase their cytotoxicity against cancer cells. Other polyphenols, including biochanin A and quercetin, had no significant effects.

The cited studies prove that curcuminoids and flavonoids are effective agents for the repression of CTC spreading because they target EMT in primary tumours. We can assume that they also suppress macrometastasis formation by targeting the activation and survival of dormant cancer cells. In addition, the formation of macroscopic metastasis is accompanied by a second EMT event [229,247], which is a possible target for curcuminoids and flavonoids. However, knowledge of this phenomenon is very limited, and the presented model of metastasis formation needs to be validated in NSCLC.

### 4.3. Effect of Curcumin and Flavonoids on the Migration of Cancer Cells

Another discussed strategy for metastasis suppression is targeting the mobility of cancer cells. At present, some high-impact studies strongly recommend targeting cytoskeletal dynamics as an optimal method to suppress metastatic activity [49].

Chen et al. reported that curcumin administration to the 801D (human large-cell lung carcinoma) cell line led to significant inhibition of EGF- or TGF-β1-induced lung cancer cell migration and invasion [276]. These inhibitory effects of curcumin were related to the inhibition of Rac1/PAK1 signalling pathways, MMP-2/9 expression, and actin cytoskeleton rearrangement. In a mouse model, curcumin (60 mg/kg) displayed a comparable effect on tumour volume and metastatic potential to cisplatin (8 mg/kg).

Flavonoid administration could lead to repression of cancer cell mobility. Incubation of A549 cells with quercetin resulted in dose-dependent disorganization of the actin cytoskeleton [277].

The incorporation of curcumin in cancer treatment regimens aimed at erasing primary tumours may aid targeting and suppress metastases. High-impact studies of animal models strongly imply a high potential of this approach. For example, the combination of cisplatin and curcumin led to a significant reduction in lymph node metastasis and primary tumour size [248]. Application of isorhamnetin in combination with cisplatin and carboplatin led to inhibition of cancer cell migration [278]. This effect was associated with the induction of microtubule depolymerization by isorhamnetin.

**Figure 4:**
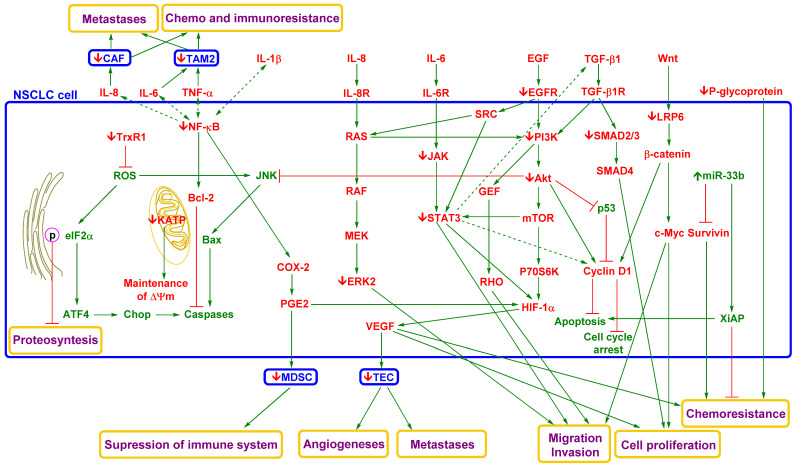
Simplified model of the effects of curcumin on NSCLC cells [128,196,242,243,244,245,248,249,276,279,280,281,282,283,284]. NSCLC is associated with dysregulation of numerous signalling and regulatory pathways. Some signalling factors (e.g., IL-6, IL-8, EGF, TGF-β1, and Wnt) produced by cancer or tumour-associated cells can induce migration and invasion phenotypes of NSCLC cells and thereby support CTC spreading. These pathways are interconnected, and dysregulation of one can induce dysregulation of another and modulate therapeutic targeting. Nevertheless, curcumin targets multiple signalling pathways to repress this phenomenon. Its effect is associated with repression of ERK2, JAK, STAT3, EGFR, PI3K, Akt, SMAD 2/3, and β-catenin. In addition, curcumin induces oxidative stress in the endoplasmic reticulum by inhibiting TrxR1 and mitochondrial-dependent and mitochondrial-independent apoptotic pathways and repressing drug resistance. Reduced levels of signalling factors produced by NSCLC cells, such as IL-6, 8, VEGF, and PGE2, lead to decreased activity of tumour-associated/tumour-infiltrating cells and thereby decreased support of NSCLC cell metastasis, proliferation, and survival in the tumour microenvironment. Curcumin’s effects on the tumour microenvironment also include activation of the immune system and suppression of angiogenesis and chemoresistance and immune resistance. Akt, protein kinase B; Bcl-2, B cell lymphoma 2; CAF, cancer-associated fibroblast; COX-2, cyclooxygenase-2; EGF, endothelial growth factor; EGFR, endothelial growth factor receptor; ERK2, extracellular signal-regulated kinase 2; GEF, guanine nucleotide exchange factor; HIF-1α, hypoxia-inducible factor 1α; JAK, Janus tyrosine kinase; JNK, c-Jun N-terminal kinase; IL-1β, interleukin 1β; IL-6, interleukin 6; IL-6R, interleukin 6 receptor; IL-8, interleukin 8; IL-8R, interleukin 8 receptor; KATP, ATP-sensitive potassium channel; LRP-6, low-density lipoprotein receptor-related protein 6; MAPK, mitogen-activated protein kinase; MDSC, myeloid-derived suppressor cell; mTOR, mammalian target of rapamycin; NF-κB, nuclear factor kappa-light-chain-enhancer of activated B cells; P70S6K, ribosomal protein S6 kinase beta-1; p-eIF2α, phosphorylated eukaryotic translation initiation factor 2 subunit 1; PGE2, prostaglandin E2; PI3K, phosphoinositide 3-kinase; ROS, reactive oxygen species; STAT3, signal transducer and activator of transcription 3; SRC, intracytoplasmic tyrosine kinase; TAM2, tumour-associated macrophage M2 phenotype; TEC, tumour endothelial cell; TGF-β, transforming growth factor beta; TGF-β1R, transforming growth factor beta 1 receptor; TNF-α, tumour necrosis factor alpha; Trx1, thioredoxin reductase 1; VEGF, vascular endothelial growth factor; ΔΨm, mitochondrial membrane potential. Green arrow = induction/activation of factor/phenomenon/cell, dotted (indirect); red arrow = repression/inhibition of factor/phenomenon/tumour-supporting cell; green factor = anticarcinogenic factor; red factor = carcinogenic factor; **↑** = curcumin activation/induction; **↓** = curcumin repression/inhibition.

In addition to suppressing cancer metastasis, curcumin also has a cytostatic effect. For example, Mirza et al. reported that curcumin administration could lead to specific killing of circulating metastatic cells [249]. The authors reported that cancer cells obtained from peripheral blood of patients with lung adenocarcinoma displayed a cancer-stem-cell-related phenotype (expression of P-glycoprotein 1 (CD44), prominin-1 (CD133), and aldehyde dehydrogenase). These cells also had strong chemoresistance; for example, gemcitabine, even at concentrations higher than 100 μM, did not cause any significant cytotoxicity. Treated cells showed a high response to a low concentration of nanoformulated curcumin (IC_50_ = 10 µM). This effect was coupled with inhibition of the DNA repair mechanism and DNA damage. In contrast, the cytotoxic effect against healthy cells (peripheral blood mononuclear cells) was lower. Possible effects of curcumin on NSCLC cells are shown in Figure 4.

In NSCLC, curcumin represses some important signalling pathways. It inhibits the activation/phosphorylation of JAK and STAT3 (part of the EGF and IL-6 signalling pathways) [285]. STAT3 is constitutively activated in approximately 50% of NSCLC primary tumours and NSCLC cell lines and is associated with poor prognosis [286,287,288]. Jiang et al. reported that in A549 cells, curcumin application led to not only decreased EGFR expression but also reduced EGFR activity via induction of ubiquitin-activating enzyme E1-like [289]. In human samples (NSCLC tumour tissues and adjacent tissues from NSCLC patients (stages I-IIIa)), there was an inverse correlation between curcumin administration and the activity of the EGFR/AKT/NF-κB pathway.

In athymic nude mice bearing NCI-H460 tumours, curcumin-induced inhibition of JAK and STAT3 phosphorylation led to reduced tumour weight and improved the survival rate of mice. In addition, STAT3-regulated promoter activation of VEGF, Bcl-xL, and cyclin D1 was also repressed after treatment [285]. Targeting STAT3 thus represents another promising approach for reducing CTC counts. Zhang et al. reported that suppression of the JAK/STAT3 pathway reduced CTC seeding in primary tumours (with a nude mouse model of human osteosarcoma) [290]. Mautsaka et al. found that the CTC EGFR expression relative to baseline was strongly associated with regorafenib resistance in patients with refractory metastatic colorectal cancer [291].

One of key factors of NSCLC pathogenesis is NF-κB. Details on its role were described by Dimitrakopoulos et al. [292]. NF-κB has been found to control inflammation, proliferation, survival, apoptosis, angiogenesis, EMT, metastasis, stemness, metabolism, and therapy resistance [293,294]. For example, Sun et al. reported that activation of the PI3K/Akt/NF-κB/tyrosine kinase B (TrkB) pathway led to resistance of cancer cells to anoikis and thereby higher metastatic activity in a mouse model of hepatocellular carcinoma [295].

An interesting approach is using microRNAs (miRNAs) as therapeutics. The striking advantage of miRNAs is that a single miRNA has the potential to simultaneously suppress several oncogenic pathways because it can target multiple genes. Naidu et al. reported targeting of the miR-23b cluster, or miR-125a-5p, which silenced KRAS and NF-κB signalling and resulted in significant repression of the tumourigenicity of CTCs from NSCLC patients in a mouse model [296]. Similarly, Lin et al. showed that TNF-α-stimulated degradation of IkappaB-alpha and translocation of NF-κB into the nucleus subsequently induce MMP-9 expression in A549 cells [297]. Nevertheless, curcumin application led to the suppression of IκB-α phosphorylation and thereby NF-κB activation [298]. Lee et al. reported that interferon alpha (IFN-α)-induced activation of NF-κB and COX-2 was inhibited by curcumin in A549 cells [299]. Accordingly, curcumin inhibition of the migratory and invasive abilities of NSCLC cells seems to be mediated through the NF-kB/MPP pathway according to studies in a mouse model [300,301].

On the other hand, CTC survival can be associated with deacetylase sirtuin 1 (SIRT1) expression, which leads to suppression of NF-κB and ROS activity in some oncological diseases, such as breast cancer [85]. However, the role of SIRT1 expression is not clear, as it can have oncogenic and antitumour effects. For example, repression of cancer cell migration and angiogenesis via activation of both intrinsic (caspase-9) and extrinsic (caspase-8) apoptotic pathways was found to be associated with SIRT1 activation by curcumin in squamous cell carcinoma of the head and neck [302]. Nevertheless, in human colon cancer cells, the repressive effect of curcumin on cell viability and migration caused inactivation of SIRT1 via covalent modification of the cysteine 67 residue [303].

A great benefit of curcumin application is its targeting of hypoxia-related metabolism in NSCLC. Cancer cells, which are located further from blood vessels due to their faster metabolism, have lower oxygen levels. Therefore, hypoxic tumours display significantly lower oxygen pressure (10 mmHg or less) [304]. Hypoxia-activated HIF-1α can stimulate carcinogenesis via induction of numerous signalling pathways (TGF-β1, EGF, Wnt, and Notch), transcription factors (Snail, Slug, Twist, and Zeb1/2) and other factors [305,306]. These effects lead to the induction of a cancer stem cell phenotype with features such as immune and drug resistance, a mesenchymal phenotype, and metastatic activity. In metastases, HIF-1α levels have been found to be significantly increased compared with those in primary breast tumours [307]. This finding suggests the importance of HIF-1α levels in CTC spreading.

Hypoxic CTCs have a greater chance of survival in the bloodstream because of their PD-L1 expression [308]. Nevertheless, the influence of hypoxia on CTC metastatic activity is complicated. Donato et al. reported that in a breast cancer model under hypoxic conditions, liberated CTC clusters contained cells with a hypoxic phenotype [48]. In contrast, in normoxia, liberated tumour cells have a normoxic phenotype. However, knockout of HIF-1α did not repress cluster formation. Moreover, knockout of VEGF (an angiogenic factor induced by HIF-1α) led to tumour shrinkage but also supported cluster formation.

It has been proven that curcuminoids could represent suitable structural motifs for targeting HIF-1α hypoxia-related signalling. For example, Li et al. reported that curcumin significantly decreased the expression of HIF-1α and VEGF in a mouse model of lung cancer (A549 cells) [301]. Ye et al. reported curcumin-induced repression of HIF-1α, which led to downregulation of P-glycoprotein expression and increased chemosensitivity of A549 cells [309]. Similarly, Fan et al. observed decreased levels of HIF1α, p-mTOR/mTOR, VEGF, and VEGFR in a mouse model of Lewis lung carcinoma [310].

An important anticancer effect of curcumin could be its repression of Smad 2/3 phosphorylation. Datta et al. reported that curcumin represses Smad 2/3 phosphorylation in TGF-β1-dependent H358 and A549 (NSCLC) cell lines [311]. However, no difference in curcumin-induced toxicity or changes in tumourigenicity were reported between the TGF-β1-dependent cells and ACC-LC-176 cells (an NSCLC line independent of TGF-β1). Nevertheless, some results obtained from studies of other cancer types imply that curcumin can inhibit TGF-β1-induced EMT, invasion, and IL-6 expression, and thereby cancer metastasis [242,312,313,314,315,316].

Targeting the Wnt/β-catenin pathway is an intensively studied method in cancer treatment. Wang found that the suppressive effect of curcumin on β-catenin expression was mostly caused by the induction of oxidative stress in NSCLC cells (A549) [237]. Lu et al. reported that administration of curcumin to NSCLC cells (95D and A549) reduced the overexpression of metastasis-associated protein 1, which caused a decrease in Wnt/β-catenin signalling (downregulation of β-catenin, cyclin D1, and MMP-7) [317]. Wen et al. reported that targeting Wnt/β-catenin signalling could lead to a reduction in CTC counts in oncology patients [318].

Flavonoids represent potent agents for the suppression of cancer spreading. They can decrease the activity and expression of numerous factors associated with NSCLC metastatic activity, such as NF-κB, STAT3, Akt, β-catenin, VEGF, HIF-1α, EGFR, and mTOR. For example, QSAR modelling strongly implies that EGCG is a potent EGFR binder [319]. Minnelli et al. reported that the interaction energies were 98, 54, and 75 kcal/mol for wild-type EGFR, T790M/L858R-mutated EGFR, and ELREA (EGFR with deletion of five amino acids in exon 19), respectively [320]. For comparison, the obtained values for erlotinib were 71 (wild-type EGFR), 39 (T790M/L858R-mutated EGFR), and 97 kcal/mol (ELREA). Liu et al. found that EGCG is a dual inhibitor of PI3Kα (IC_50_ = 0.69 µM) and mTOR (IC_50_ = 0.12 µM), an inhibitor of EMT, and can overcome gefitinib resistance [321]. Zhang et al. found that EGCG not only reduces the active form of NF-κB but also directly impacts this factor (K_D_ = 4.8 × 10^−5^ M) [322]. Rawangkan et al. reported that EGCG pretreatment of Lu99 cells (an NSCLC cell line) strongly decreased PD-L1 expression and induction by EGF and IFN-γ [323].

Both EGFR mutations and PD-L1 overexpression enhance CTC and metastatic cell spreading in NSCLC. CTCs with EGFR mutations and PD-L1-expressing CTCs have been shown to correlate with shortened OS, disease progression, formation of metastases, and therapeutic failure (Section 3.1 and Section 3.2). As such, agents with low toxicity, such as flavonoids, could represent promising tools for decreasing CTC counts and thereby suppress metastasis.

The application of flavonoids can, via various independent mechanisms, repress NSCLC cell survival, proliferation (e.g., modulation of cyclin-dependent kinases, caspase induction, activation of apoptotic factors, and repression of survival) and migration; for example, flavonoids can inhibit MMPs. Suzuki et al. described EGCG binding with the β- and γ-chains of fibrinogen, which represses fibrinogen interaction with NSCLC cancer cells (e.g., LL2-Lu3 cells) and thereby impairs their metastatic spread [324]. More details regarding the effects of flavonoids on NSCLC and lung cancer models are described in Table 4.

Although curcumin and flavonoids display various toxic effects on cancer cells, they are surprisingly less toxic to normal cells. A possible explanation could be their effect on the redox homeostasis of cells [349]. Higher ROS levels are strongly associated with characteristics of carcinogenesis, such as increased mutations and dysregulation of signalling cascades (MAPK, PI3K/Akt, Nrf2, AP-1, NF-κB, STAT3, and p53) [350,351]. Curcumin and flavonoids are potent antioxidants and can protect normal cells against the carcinogenesis and apoptosis induced by ROS. On the other hand, ROS generation is part of numerous therapeutic strategies [351,352], and the application of antioxidants can be counterproductive. Some clinical trials have found that β-carotene and retinol can promote tumour growth and metastasis in cancer patients [353,354]. Godman et al. found that their application is associated with a higher risk of NSCLC in female patients [354].

However, hypermetabolism of cancer cells results in higher production of ROS and antioxidant capacity. Curcumin dysregulates redox balance by disrupting mitochondrial homeostasis via oxidative stress. It causes opening of the mitochondrial permeability transition pore, mitochondrial swelling, loss of mitochondrial membrane potential, and inhibition of ATP synthesis [355,356]. Curcumin can also supports the mitochondrial apoptotic pathway by inducing overexpression of pro-apoptotic Bax protein and reduced expression of Bcl-2 in NSCLC cells [357]. Moreover, the cytotoxic effect of curcumin or its analogues can be detected via the accumulation of curcuminoids in the ER and the upregulation of the ER stress-related unfolded protein response, which leads to inhibition of protein synthesis and cell cycle arrest [357,358]. In addition, curcuminoid application leads to increased intracellular ROS levels and increased SOD and γ-GCS activity [237]. Additionally, flavonoids such as fisetin can induce oxidative stress in cancer cells [336].

Another possible explanation for the high cell selectivity of curcuminoids and flavonoids is their effects on cellular hypoxia. These agents can significantly induce higher toxicity in hypoxic cancer cells [359,360]. However, in the case of normal cells, their application has protective effects and leads to restoration of normal metabolism [360,361]. For example, in ischaemic muscles (which have a decrease in oxygen level due loss of blood flow), curcumin helps tissue restoration [310].

Cancer cells have higher concentrations of and need iron ions [362]. Targeting iron homeostasis with flavonoid chelators such as quercetin has been intensively studied in anticancer treatment and is usually highly specific for cancer cells [363]. The anticancer effects of curcuminoids are also associated with iron chelation [364,365]. Nevertheless, higher levels of transition metal ions can lead to higher levels of flavonoids and curcumin metal complexes. These complexes also likely have their own biological/anticancer activities [366]. For example, Tan et al. found that the anticancer effect of iron−flavonoid complexes is associated with DNA targeting [367]. Chen et al. reported that hyperoside activity against hypoxic A549 cells was significantly higher in the presence of iron ions [328]. Similarly, iron−curcumin complexes (IC_50_ = 8 μM) displayed higher cytotoxicity against MDA-MB-231 breast cancer cells than curcumin (IC_50_ = 24 μM) [368].

In addition, antimetastatic effects can be achieved by using a noncytotoxic dose [236,369]. Tabasum et al. observed that fisetin (10 μM) decreased the expression of NF-κB, STAT3, and β-catenin, and repressed EMT in A549 and H1299 cells [236].

The above findings imply that curcumin and flavonoids are prospective agents for incorporation in NSCLC therapy with effective targeting against cancer cell migration. However, oncogenic signalling pathways display strong redundancy; therefore, inhibiting one signalling pathway may not be enough. Incorporating multiple targeted agents, such as curcuminoids, flavonoids, or their combination, in therapeutic regimens could effectively avoid this phenomenon. This strategy could control the growth of tumours or shrink their volume with a reduced risk of metastasis. Many high-impact studies have shown that these compounds are potential agents for combination therapy [370,371,372,373,374]. Their application can significantly increase the efficiency of classically used therapies (e.g., chemotherapy, TKIs, immunotherapy, and radiotherapy) and repress tumour resistance. However, the anticancer effects of curcuminoids and flavonoids discussed above were mostly seen in in vivo and in vitro studies. A meaningful assessment of their potential therapeutic effects is not possible without other clinical trials.

## 5. Future Directions

CTCs are strong markers for disease development and prognosis and can be used in the design of anticancer regimens, especially in combination with proteomic, transcriptomic, and genomic profiling of metastatic CTCs [32,60]. However, the spread of CTCs is not an isolated phenomenon and is influenced by numerous factors, such as tumour-associated cells (e.g., M2 TAMs, MDSCs, Treg cells, CAFs, and TECs) [138,139]. Therefore, determining the CTC level or better analysing circulating tumour-associated cells that can strongly boost CTC metastatic ability or drug resistance could provide more precise information for future therapeutic development and design. Some clinical studies have shown that higher levels of tumour-associated cells are associated with worse therapy prognoses [146,153,157,176,200]. Clusters of CTCs in combination with tumour-associated cells, and not single CTCs, are likely associated with increased risk [8,140]. In accordance with this hypothesis, CTC clusters have been observed in NSCLC patients [95,375]. However, new analytical methods that are significantly easier and cheaper and have higher sensitivity are needed. For example, Watanabe developed the On-chip Sort method (22/30; median 5; range 0–18 cells/5 mL blood), which demonstrated significantly higher CTC-capturing ability for patients with metastatic NSCLC than CellSearch (9/30; median 0; range 0–12 cells/7.5 mL) (*p* < 0.01) [376]. Hosokawa et al. also developed a microcavity assay (MCA) system that could isolate more CTCs and CTC clusters from NSCLC patients than the CellSearch system (Veridex LLC, Raritan, NJ, USA) [375].

CTC clusters are very strong metastatic forms of CTCs, implying their importance for clinical diagnosis [8,24,140]. Nevertheless, and despite unquestionable progress in this field, their identification and analysis remain a great challenge. Hasan et al. reported that CTC clusters from a mouse model of prostate cancer have cells with an epithelial surface phenotype and mesenchymal core phenotype [377]. In addition, CTCs can also form heterotopic clusters with tumour-associated cells, such as CAFs, which induce higher survival in the bloodstream [135]. On the other hand, although the determination of CTC count or CTC phenotype is complicated, an FDA-approved system for the determination of CTC count is already being used. Therefore, at present, determination of CTC count and CTC analysis have high utility in clinical diagnosis.

EpCAM is robust, reliable, reproducible standardised kit, which enables semiautomated processing and stain with one further antibody [378,379]. At present, it is FDA-approved for diagnostics in breast, prostate, and colorectal cancer. On the other hand, some limitation regarding its application in routine diagnostics can be noted. EPCAM is very expensive, has a low capture of CTC without epithelial markers (e.g., undergone EMT), and numbers of CTCs detected in the blood sample of NSCLC patients are sometimes lower than other kits (e.g., ISET) and cannot be used for the detection of CTC clusters [379]. ISET is less expensive and enables determination of CTC clusters; however, it captures small CTC (under 8 μM) poorly, and manual processing limits its robustness, although it has potential for automatization.

Due to their importance in tumour metastasis, CTCs are valuable targets in anticancer therapy. Curcuminoids and flavonoids are promising agents for decreasing CTCs, as well as CTC clusters and support cells, in NSCLC therapy. In addition to their own highly selective cytostatic effects, these agents display strong potential for the repression of metastatic spread by various independent mechanisms [139]. However, their efficacy, especially curcumin, can be strongly limited by low solubility and biostability [380]. Nevertheless, this type of problem can be effectively solved by drug delivery systems [381,382,383]. In the case of curcuminoids and flavonoids, various nanoparticles or curcumin nanofibres have showed high efficacy in tumour targeting and metastatic suppression [370,384,385]. For example, Su et al., using a mouse model of lung cancer, designed and tested curcumin-modified silica nanoparticles for inhalation therapy [386]. The anticancer efficacy of a strategy inducing IL-6 and metastatic suppression was significantly higher than that of curcumin alone.

Incorporating these novel agents in therapy could lead to new neoadjuvant and adjuvant strategies. Classical neoadjuvant therapeutic regimens aim to shrink tumours using drugs that target cancer cells (mainly cytostatic drugs) and only indirectly affect metastasis-initiating cells [6]. However, the in vitro-determined drug sensitivity of CTCs suggests that agents related to CTCs have potential to optimise therapy [387]. Most deaths from cancer, including NSCLC, are not caused by primary tumours but by metastasis. [5] Therefore, a new therapeutic method that focuses on inhibiting metastasis formation via the use of migrastatic drugs (inhibitors of cell migration) instead of reducing the tumour mass has been proposed [49,50]. For example, some androgen receptor inhibitors (e.g., apalutamide, enzalutamide, and darolutamide) can delay metastases in high-risk nonmetastatic castration-resistant prostate cancer [51]. In NSCLC, the application of TKIs or ICIs and antibodies against IL-6R can suppress metastasis formation [52,53,54]. Nevertheless, NSCLC displays high heterogeneity, and multifunctional agents have a better chance of avoiding the development of resistance. In addition, potential therapeutic strategies and agents are still lacking.

**Figure 5:**
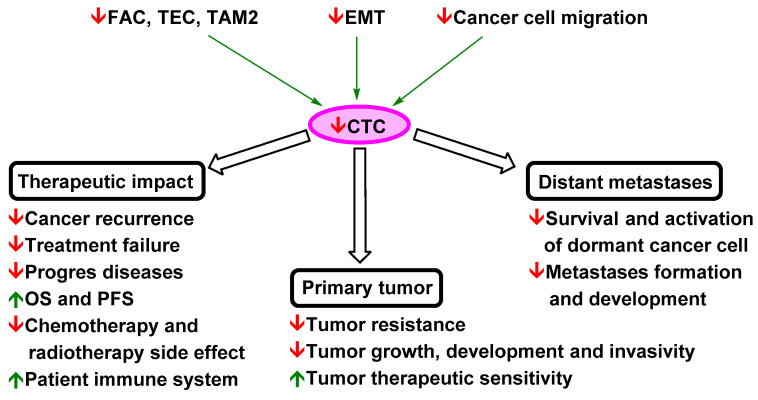
Curcuminoids and flavonoids in migrastatic and cytostatic (MICY) therapy. Green arrow = induction/activation of CTC; **↑** = curcuminoids/flavonoids activation/induction; **↓** = curcuminoids/flavonoids repression/inhibition.

Incorporating low-toxicity agents such as curcuminoids and flavonoids into chemotherapy regimens could effectively combine cytostatic and migrastatic effects to form a new migrastatic and cytostatic (MICY) combination strategy. The basic philosophy of the MICY combination (isolation and destruction) is to reduce the tumour mass and block the spread of CTCs and other circulating tumour-associated cells (Figure 5). MICY could also be applied after surgical eradication of primary tumours to target micrometastases.

Curcuminoids and flavonoids can be recommended for this purpose for several reasons. They have low toxicity and are part of daily dietary uptake [388]. Therefore, they should be able to be used daily and long-term, perhaps for the entire life of the patient. These agents repress the activation (Wnt/β-catenin signalling [236,237]) and survival (hypoxia [239,240]) of dormant cancer cells. They are potent antioxidants [232,233,234,235] and EMT inhibitors [223,224] and therefore can also suppress micrometastasis survival and macrometastasis formation [234,236,252]. Their administration can repress the recruitment and activation of tumour-associated cells that strongly support metastasis formation and development [150,154,158,159,173,174,175,185,189]. They are multifunctional agents; therefore, their risk of resistance is significantly lower. Additionally, combination can significantly improve their efficacy (e.g., by reducing multidrug resistance) [185,389,390,391,392,393,394,395]. These potential benefits have led to the administration of combined rather than single agents, and we expect that this approach will lead to a significantly lower risk of resistance.

The high potential of these agents in anticancer therapy has also been shown in numerous clinical trials (see Table 5). Their incorporation into therapeutic regimens led to a decrease in oxidative and inflammatory stress, a decrease in the level of metastatic factors, suppression of therapy side effects, and improvement of patient quality of life. In addition, some of the studies imply that such applications can have protective effects for subjects with higher cancer risk. The above clinical trials include various limitations and used various therapeutic regimens; as such, the usability of the acquired knowledge in the treatment of lung cancer may be limited. Therefore, other clinical trials are highly awaited to determine the therapeutic effects of these strategies in NSCLC patients.

The question is whether the reduction of CTCs, as well as CTC clusters, and thereby cancer metastasis in NSCLC, has utility. Some factors (e.g., TGF-β, EGF, IL-6, IL-8, and IL-1β) that are decreased by curcuminoid or flavonoid application have been found to play a significant role in CTC formation and metastatic activity (Section 4.3).

Some studies imply that these agents have promising potential to reduce CTCs. Ried et al. reported that the application of a nutrient combination (curcumin, garlic, green tea, grape seed extract, modified citrus pectin, and medicinal mushroom) decreased the CTC count in patients with oncological diseases [203]. Similarly, Pang et al. found in a mouse breast cancer model that the application of hesperidin (30 mg/day) decreased the metastasis number and CTC count by approximately half [204].

In addition, the decrease in d-dimer (a product of fibrin degradation and the end product of coagulation activation) and thrombin (conversion of fibrinogen to fibrin) generation induced by isoquercetin [422] also suggest an effect against CTCs. Kirwan et al. reported that in metastatic breast cancer, hypercoagulability markers (d-dimer) are associated with higher lethality (44% versus 19%; 1 year after the start of the study) for patients with CTCs (> 1) [425]. In breast cancer and glioblastoma cell lines, thrombin induces higher proliferation, expression of angiogenetic proteins (Twist and Gro-α), and migration [426]. Levitan et al. found that thromboembolic diseases are strongly associated with malignancy (in 90% of patients with metastasis) [427].

Another consideration is the effects of curcuminoids and flavonoids on the immune system. Basak et al. reported that the application of turmeric extracts (curcuminoids) led to an increase in CD4+ T and CD8+ T cells in the tumour tissue of patients with oral cancer [410]. Similarly, curcumin was found to induce the conversion of Treg cells into Th1 cells and increase IFN-γ in patients with lung or colon cancer [154,216]. In breast cancer patients, higher Treg cell levels were associated with higher CTC abundance, and higher CD8+ T cell and IFN-γ levels negatively correlated with CTC count [199,201].

The above results suggest that curcuminoids and flavonoids are potential agents for targeting CTC spread and metastasis formation. Nevertheless, MICY therapy design is still in the preliminary stages, and numerous animal and clinical studies are needed for its evaluation.

## 6. Conclusions

CTCs are a potential biomarker for NSCLC diagnosis and play key roles in tumour spread. Numerous clinical studies have shown a negative correlation between the level of CTCs in blood and therapeutic prognosis indicators (mainly OS and PFS). This review shows the potential utility of CTC parameters for obtaining information, such as protein expression and gene mutations (PD-L1 and EGFR, respectively), about primary tumours and metastases and the applicability of such information for the management and design of therapy. The effects of these promising, low-toxicity natural agents (curcuminoids, mainly curcumin and flavonoids) with mechanisms affecting the genesis and spread of cancers were presented and discussed. The cited high-impact studies imply high potential and applicability of CTC targeting in NSCLC treatment.

## Figures and Tables

**Table 2:** Analysis of CTC count in patients treated with agents targeting programmed cell death protein ligand 1 (PD-L1) and epidermal growth factor receptor (EGFR). TKI, tyrosine kinase inhibitor.

Patient Characteristics	Clinical Finding	Ref.
104 patients with stage IIIB or IV disease; agents targeting PD-L1 or PD-1, 6 weeks (The Netherlands)	≥1 (32%) ^1^	[89]
	CTC count for prediction ^2^ (baseline): ≥1; (OS (12.1 vs. 4.5) and PFS (4.8 vs. 1.4)	
7.5 mL, CellSearch (Veridex LLC, Raritan, NJ, USA)		
68 patients with stage IIIB or IV disease; first-line TKI treatment failure, EGFR-T790M (China)	CTC distribution: ≥5 (75%)	[104]
	CTC count for prediction (baseline): ≥5; PFS (9.3 vs. 6.5)	
7.5 mL, CellSearch (Veridex LLC, Raritan, NJ, USA)		
107 patients with stage IIIB (ineligible for sequential radiotherapy or concurrent chemo/radiotherapy) or stage IV disease; erlotinib/gefitinib therapy, 28 days ^3^ (China)	CTC distribution: ≥2 (44%) and ≥5 (15%)	[118]
	CTC count for prediction (baseline): ≥5; PFS (11.1 vs. 6.8)	
7.5 mL, CellSearch (Veridex LLC, Raritan, NJ, USA)		
41 relapsed or refractory NSCLC patients; erlotinib/pertuzumab, 3 weeks (USA)	CTC distribution: ≥1 (78%) and ≥5 (42%)	[105]
	Agreement (cDNA, tumour biopsy) of EGFR and KRAS mutations was not observed between CTCs and tumour tissues	
CellSearch; EGFR status was determined by immunofluorescence; mutations in EGFR, KRAS, PIK3CA, BRAF, NRAS, and AKT1 were assessed by DxS kits and TaqMan genotyping assays (Qiagen, Venlo, Netherlands)		
37 patients with stage IIIB or IV disease; no previous chemotherapy, EGFR mutations (Italy)	CTC distribution: ≥1 (13%)	[100]
	84% ^4^ had EGFR mutations, 81% had in-frame deletions (exon 19), 19% had point mutations (exon 21), 13% had multiple mutations, 94% had mutations in tumour tissue	
7.5 mL of peripheral blood was used for CellSearch analysis, PCR amplification (MIDs), and next-generation sequencing (massively parallel pyrosequencing)		
40 patients with stage III recurrent disease following locoregional treatment who developed resistance to a primary EGFR TKI, 30 days (USA)	76% were suitable for genotyping, 57% of CTC samples had T790M mutation, 74% had biopsy agreement (CTC and tumour biopsy agreement)	[103]
10 mL of blood was used for HbCTC-Chip (EpCAM) assessment of specific T790M amplification		
10 patients with the EGFR DelEx19 mutation (Germany)	Low mutation burden (40% of patients) delayed treatment failure (116 vs. 355 days)	[111]
20 mL of peripheral blood was used for assessment with anti-EpCAM (CD326, positive) and anti-CD45 (negative) microbeads (Miltenyi Biotech, Bergisch Gladbach, Germany), real time-PCR, and melting curve analysis		

^1^ Proportion of patients with a given number of CTCs. ^2^ OS and PFS shown in months. ^3^ Length of the therapy. ^4^ Proportion of CTC samples/patients.

**Table 3:** Effects of curcuminoids and flavonoids on tumour-associated cells.

Agents	Model	Effects	Lit.
Phytosomal curcumin	Immune-competent syngeneic C57BL6 mice with orthotopically implanted mouse GL261 (GBM) cells	TAM phenotype (↓STAT3 ↓IL10, ↑IL12, ↑STAT1, ↓ARG1 and ↑MCP-1), ↑NK recruitment and ↑TAM repolarization from M2 to M1	[149]
TriCurin	Mice implanted with UMSCC47 (HNSCC) cells	TAM phenotype (↓ARG1, ↓IL10, ↑iNOS, ↑IL12, ↓STAT3, ↑STAT1 and ↑NF-KB), ↑NK recruitment and ↑TAM repolarization from M2 to M1	[150]
Curcumin	Mice implanted with GL261 (GBM) cells	Microglia phenotype (↑iNOS, ↓ARG2 and ↑NF-kB)	[205]
Pro-EGCG	Mice implanted with AN3CA and RL95-2 (EC) cells	↓VEGFA ↓HIF1α, ↓SDF1 and ↓TAM infiltration	[206]
EGCG exosomes	Mice implanted with 4T1 (BC) cells	TAM phenotype (↓IL-6, ↓TGF-β, and ↑TNF-α), ↓CSF-1, ↓CCL-2, ↓tumour growth and ↑TAM repolarization from M2 to M1	[207]
Curcumin	Mice implanted with HepG2 (HC) cells	(MDSC inducers (↓GM-CSF and ↓G-CSF), ↓MSDC phenotype (↓TLR4/NF-κB), ↓IL-6, ↓IL-1β, ↓PGE2, ↓COX-2, ↓VEGF and CAF marker (↓CD31 and ↓αSMC)	[208]
Curcumin	Mice implanted with Lewis lung carcinoma cells	↓IL-6 and ↓MDSCs	[158]
Curcumin	Mice implanted with 4T1 (BC) cells	↓G-MDSC and ↑M-MDSC polarization to M1 TAMs, ↑CD4+ T cells and ↑CD8+ T cells	[209]
Curcumin−PEG conjugate	Mice implanted with B16F10 (melanoma) cells	↓Treg cells ↓MDSC and CAF markers (↓α-SMA and ↓CD31)	[210]
Lipid-based Trp2 peptide combination vaccine		↑CD4+ T cells and ↑CD8+ T cells	
Curcumin	Mice implanted with OSCC (induced by 4NQO) cells	↑CD8+ T cells, ↓Treg cells and ↓MDSCs	[159]
Bisdemethoxycurcumin	Immunocompetent mice implanted with subcutaneous or lung metastasised MB79 (bladder cancer) cells	↑CD8+ T cells, ↓Treg cells and ↑IFN-γ	[162]
α-PD-L1 antibody combination		↓MDSCs and CD8+ T cells (↑IFN-γ, ↑granzyme B, ↑perforin and ↓exhaustion)	
Quercetin	Human and mouse G-MDSCs	↑ESR/STAT3, ↑NOS2 and prolonged MDSC survival in mice	[211]
EGCG	M-MDSCs	↓Arg-1/iNOS/Nox2/NF-κB/STAT3, ↓IL-6, ↓IL-10, ↓TGF-β, ↓GM-CSF, and ↑apoptosis	[212]
	Mice implanted with 4T1 (BC) cells	↓MDSCs ↑CD4+ T cells ↑CD8+ T cells	
Polyphenon E	Transgenic TH-MYCN mice	↓MDDCs	[213]
	NOD/SCID mice implanted with SHSY5Y (neuroblastoma) cells	0MDSCs	
	A/J mice implanted with syngeneic Neuro 2A (neuroblastoma) cells	↓MDSCs	
	MDSCs	↑G-CSF, ↑IL-6, ↓Treg cell induction	
Curcumin	Coculture of a primary BC line + T cells	↓TGF-β, ↓Treg cell phenotype induction (IL-2Rα, IL-6, and FoxP3) in CD4+ T cells	[214]
	Mice implanted with 4T1 cells	↓Treg cell phenotype (CD4+, CD25+, and FoxP3+)	
Curcumin	HNSCC tissue	↓CCL22 (Treg cell mobility)	[215]
Curcumin	Patients with colon cancer	↑Conversion of Treg cells into Th1 cells and ↑induction of a Th1 cell phenotype (↓FoxP3 and ↑IFN-γ)	[216]
Curcumin	Patients with lung cancer	↑Conversion of Treg cell into Th1 cells and ↑induction of a Th1 cell phenotype (↓FoxP3 and ↑IFN-γ)	[154]
Curcumin	Primary TSCC CAFs	↓α-SMA, ↓TGF-β1, ↓SDF-1, ↓MMP-2, ↓SMAD2/3, ↓Cal27, and ↓proliferation	[173]
Curcumin	Mice implanted with Cal 27 (TSCC) cells	↓α-SMA and ↓Ki67	
Curcumin	CAFs cocultured with Capan-1 and Panc-1 (pancreatic carcinoma) cells	↓CAF phenotype (↓α-SMA and ↓vimentin), ↑E-cadherin, ↓EMT and ↓cancer cell migration	[174]
	nu/nu nude mice implanted with Panc-1 tumour cells	↓Lung metastasis	
Curcumin	CAFs cultured with prostate cancer pC-3 cells	↓ROS, ↓IL-6, ↓CXCR4 and ↓MAOA/mTOR/HIF-1α	[217]
Curcumin	Primary breast CAFs	↓α-SMA, ↓JAK2/STAT3, ↓SDF-1, ↓IL-6, ↓MMP-2 and ↓MMP-9, ↓TGF-β and ↓migration ability	[218]
Curcumin	TNF-α-activated ECs	↓NF-κB, ↓adhesion molecules (↓ICAM-1 and ↓VCAM-1) and ↓monocyte adhesion	[189]
Curcumin and EGCG or both agents combined	Coculture of ECs with SW620, HCT116, and HT-29 (CC) cells	↓TEC transition, ↓TEC phenotype (↓JAK, ↓STAT3, ↓IL-8, ↓TEM1, ↓TEM8 and ↓VEGFR2) and ↓TEC migration	[185]
	Mice implanted with patient-derived CCs	↓JAK, ↓STAT3 and ↓IL-8	

α-SMA, alpha-smooth muscle actin; ARG1, arginase 1; ARG2, arginase 2; CD31, a platelet endothelial cell adhesion molecule; COX-2, cyclooxygenase-2; G-CSF, granulocyte colony-stimulating factor; ESR, oestrogen signalling receptor; GM-CSF, granulocyte−macrophage colony-stimulating factor; iNOS, inducible nitric oxide synthase; IL-1β, interleukin 1β; IL-2Ra, interleukin 2 receptor alpha; IL-6, interleukin 6; IL-8, interleukin 8; Il-L-10, interleukin 10; IL-12, interleukin 12; ICAM-1, intercellular adhesion molecule 1; JAK, Janus tyrosine kinase; HIF-1α, hypoxia-inducible factor 1α; NOS2, NADPH oxidase 2; nitric oxide synthase 2; PGE2, prostaglandin E2; ROS, reactive oxygen species; TGF-β, transforming growth factor beta; TEM1, tumour endothelial marker 1; TEM8, tumour endothelial marker 1; TLR4, Toll-like receptor 4; STAT3, signal transducer and activator of transcription 3; SDF-1, stromal-cell-derived factor 1; VCAM-1, vascular cell adhesion molecule 1; VEGF, vascular endothelial growth factor; VEGFR2, vascular endothelial growth factor receptor 2; 4NQO 4-nitroquinoline-1-oxide; G-MDSCs, granulocytic MDSCs; M-MDSCs, monocytic MDSCs; BC, breast carcinoma; CC, colorectal carcinoma; EC, endometrial carcinoma; GBM, glioblastoma; HC, hepatocellular carcinoma; HNSCC, head and neck squamous cell carcinoma; OSCC, oral cavity squamous cell carcinoma; TSCC, the squamous cell carcinoma. **↑** curcuminoids/flavonoids activation/induction; **↓ **= curcuminoids/flavonoid repression/inhibition; **0** = without change.

**Table 4:** Examples of flavonoid effects on NSCLC models.

Flavonoid	Model	Effects	Ref.
Atalantraflavone	A549 and 95D cells	↓Vimentin, ↓N-cadherin ↑E-cadherin, ↓Twist1, ↓cell migration, ↓colony formation and ↑cisplatin sensitivity	[325]
Quercetin	A549 cells	↓Vimentin, ↓N-cadherin and ↓microtubular network	[277]
Quercetin-3 orutinoside	BALB/c nude mic -bearing A549 tumours	↓Akt, ↓mTOR and ↓VEGF	[326]
Hyperoside	A549 cells	↑ERK/1/2, ↓LC3-I and ↑LC3-II ↓Akt, ↓mTOR, ↓p70S6K and ↓4E-BP1	[327]
Hyperoside	A549 cells	↑AMPK, ↑HO-1, ↓survival and ↓proliferation of cells with a hypoxia phenotype	[328]
Quercetin	A549 and H460 cells	↓Akt, ↑DR 5, ↓survivin and ↑TRAIL sensitization	[329]
Quercetin	A549 cells	↓NF-κB and ↓STAT3	[330]
Quercetin	HCC827 cells	↓IL-6-induced activation of NF-kB and STAT3, ↓colony formation, ↓migration and ↓invasion	
Quercetin	Mice implanted with HCC827 cells	↑E-cadherin, ↓N-cadherin and ↓tumour growth	[331]
Quercetin	1299 and H460 cells	↓NF-κB, IKKα, ↑I*κ*B*α*, ↑FAS, ↑TRAILR, ↑MEKK1, ↑MEK4, ↑JNK, ↑GAD45 and ↑p21^cyp^	[332]
Quercetin	A549 and HCC827 cells	↑E-cadherin, ↓N-cadherin, ↓vimentin (↓Snail, ↓Slug, and ↓Twist), ↓MMP-1, ↓MMP-2, ↓MMP-7, ↓MMP-9 and ↓MMP-12	[224]
Rhamnetin	NCI-H1299 and NCI-H460 cells	↑miR-34a ↓Notch1, ↓NF-κB, ↓vimentin, ↓N-cadherin, ↓survivin, ↓cIAP1, ↓cellular migration and ↑radiosensitivity	[333]
Isorhamnetin	A549 cells	↓LC3-I and ↑LC3-II protein	[334]
Fisetin	A549 cells	↓COX-2, ↓MMP-2/9, ↑CDKN1A/B, ↑CDKN2D, ↑E-cadherin, ↓c-myc, ↓cyclin-D1 and ↓CXCR-4	[335]
Fisetin	A549 and H1299 cells	↑E-cadherin, ↓vimentin, ↓N-cadherin, (H1299), ↓ZO-1 (H1299) ↓MMP-2, stemness markers (↓CD44 and ↓CD133), ↓β-catenin, ↓NF-κB, ↓EGFR, ↓STAT3 and ↑erlotinib (a TKI) sensitivity	[236]
Fisetin	A549 cells	↓Akt, ↓mTOR, ↓p70S6K1, eIF-4E, 4E-BP1, ↓mTOR signalling molecules (↓Rictor, ↓Raptor, ↓GβL and ↓PRAS40), ↑AMPKα, ↑pTSC2, ↓p85 and ↓p110	[336]
Fisetin	HCC827 and HCC827-ER cells	↓Akt, ↓pMAPK, ↑caspase 3/8, ↑cytochrome C, ↓AXL, ↓Snail, ↑E-cadherin and ↑erlotinib sensitivity	[337]
Fisetin	A549 cells	↓NF-κB, ↓c-Fos, ↓c-Jun, ↓ERK1/2, ↓MMP-2/9, ↓u-PA, ↓adhesion, ↓invasion and ↓migration,	[338]
EGCG	A549 and H1299 sphere cells	↓β-catenin, ↓CLOCK (↓CD133, ↓CD44, Sox2, ↓Nanog, and ↓Oct4 protein) and ↓sphere formation	[81]
	Mice implanted with A549 sphere cells	↓CLOCK (↓CD133, ↓CD44, Sox2, ↓Nanog, and ↓Oct4 protein) and ↓Ki-67	
EGCG	A549 and H1299 cells	↓NF-κB (↓BCL2, ↓BCL-XL, ↓COX-2, ↓TNF-α, ↓cyclinD1, ↓C-↓MYC, ↓TWIST1, and ↓MMP-2)	[322]
	Balb/c athymic nude mice implanted with resected patient tumour cells	↓NF-κB, ↓tumour volume, ↓Ki-67, ↓EMT	
EGCG	HCC827-Gef cells	↓PI3Kα and ↓mTOR, ↓EMT, ↓colony formation and ↑gefitinib sensitivity	[321]
EGCG	H1299 and A549 cells	↑LKB-1, ↑AMPK (↓mTOR, ↓P70, and ↓4EBP1) and ↓cell migration	[339]
EGCG	AXL-high population of H1299 spheres	↓AXL receptor tyrosine kinase, ↓ALDH1A1 and ↓Slug	[340]
	Mice implanted with an AXL-high clone of spheres	↓p-AXL, ↓ALDH1A1, ↓Slug, and ↓tumour volume	
EGCG	A549 and NCI-H460 cells	↑ROS, ↓ERK1/2, ↑CTR1 and ↑NEAT1	[341]
EGCG	A549 xenografts	↓CD31, ↓αSMA, ↓collagen IV, ↓tumour hypoxia	[342]
EGCG	Lu99 cells	↓EGF and IFN-γ-induced PD-L1 expression, ↓JAK, ↓STAT1 and ↓Akt	[323]
	A/J mice implanted with NNK-induced tumour cells	↓PD-L1, ↑IL-2 expression by tumour-specific CD3+ T lymphocytes and ↓tumour volume	
EGCG	A549 cells	↑Nrf2, ↓Keap1, ↑HO-1, ↑ROS, ↑RNS, ↓Bcl-2, ↑Bax, ↑Bak, ↑Bim ↑Puma, ↓ΔΨ_m,_ and ↓EMT	[343]
EGCG	Tumour spheres (from A549, 460, and 1299 cells)	↓NEAT1, ↑CTR1 ↓CD44+ Sox2, ↓Nanog, and ↓Oct4 protein	[344]
EGCG	A549 cells	↓Nicotine-induced effects (↓HIF-1α, ↓VEGF, ↓COX-2, ↓Akt, ↓ERK1/2, ↓vimentin, and ↑E-cadherin)	[345]
EGCG	Mice implanted with A549 cells	↓HIF-1α and ↓VEGF,	[346]
EGCG	A549 cells	↓TGF-β1 induced activation of Smad2 and Smad3, ↑E-cadherin, ↓N-cadherin, ↓vimentin, ↓HAT, ↓EMT and ↓cell migration	
EGCG	A549 and NCI-H460 cells	↓HIF-1α, ↓HPV-16 E6 and E7 oncoproteins, ↓VEGF, ↓IL-8, ↓Akt and ↓CD31	[347]
EGCG	Mice implanted with A549 cells	↓HIF-1α, ↓VEGF, ↓HPV-16 E6 and ↓E7 oncoproteins and ↓CD31	
EGCG	A549 cells	↓IGF-1 induced expression of HIF-1α and VEGF	[348]

4E-BP1, translation repressor protein; ALDH1A1, aldehyde dehydrogenase 1; AMPK, AMP-activated protein kinase; Akt, protein kinase B; Bcl-2, B cell lymphoma 2; Bcl-xL, B cell lymphoma-extra large; CTR1, copper transporter 1; Bim, Bcl-2-interacting mediator of cell death; COX-2, cyclooxygenase-2; DR5, death receptor 5; FAS, fatty acid synthase; GADD45, cell cycle inhibition growth arrest and DNA-damage-inducible 45; cIAP1, cellular inhibitor of apoptosis protein-1; CDK1/AB, cyclin-dependent kinase inhibitor 1 A/B; CDK2D, cyclin-dependent kinase inhibitor; CLOCK, clock circadian regulator; eIF2α, eukaryotic translation initiation factor 2 subunit 1; CXCR-4, C-X-C chemokine receptor type 4; EGFR, endothelial growth factor receptor; ERK1/2, extracellular signal-regulated kinase 1/2; HIF-1α, hypoxia-inducible factor 1α; HAT, histone acetyl transferase; HO-1, haeme oxygenase 1; IκBα, inhibitor of kappa B alpha; IKKα, IκB kinase α; IFN-γ, interferon gamma; IGF-1, insulin-like growth factor-1; JAK, Janus tyrosine kinase; JNK, c-Jun NH2-terminal kinase; LC3-I/II, microtubule-associated protein 1A/1B-light chain 3 I/II; Keap1, Kelch-like ECH-associated protein 1; mTOR, mammalian target of rapamycin; MEKK1, mitogen-activated protein kinase kinase kinase 1; MMP, matrix metalloprotease; NEAT1, nuclear paraspeckle assembly transcript; 1 NF-κB, nuclear factor kappa-light-chain-enhancer of activated B cells; Notch 1, Notch homologue 1; Nrf2, nuclear factor erythroid 2-related factor 2; P70S6K, ribosomal protein S6 kinase beta-1; PRAS40, proline-rich Akt substrate of 40 kDa; Puma, p53 upregulated modulator of apoptosis; RNS, reactive nitrogen species; ROS, reactive oxygen species; STAT3, signal transducer and activator of transcription 3; TNF-α, tumour necrosis factor alpha; TRAILR, tumour-necrosis-factor-related apoptosis-inducing ligand receptor; TWIST1, Twist-related protein 1; uPA, urokinase plasminogen activator; VEGF, vascular endothelial growth factor; αSMA, α-smooth muscle actin; ΔΨm, mitochondrial membrane potential; **↑ **= flavonoids activation/induction; **↓ ** = flavonoids repression/inhibition.

**Table 5:** Curcuminoids and flavonoids in clinical trials.

Agents/Doses	Subjects	Clinical Findings	Ref.
Meriva (Thorne Research Inc., Dover, ID, USA; curcuminoid, 180 mg/day), 8 weeks	Oncological patients (solid tumours)	↑QoL, ↓TNF-α, ↓TGF-β, ↓IL-6, ↓substance P, ↓hs-CRP, ↓CGRP, ↓MCP-1, ↓IL-8 (placebo was more effective), negative correlation between QoL and TGF-β	[119]
Capsule curcuminoid powder (1440 mg/day), six months	Prostate cancer patients treated with intermittent androgen deprivation therapy	↓Progression (10.3% vs. 30.2%), 0PSA, 0testosterone level, 0HRQOL, ↓adverse drug reaction	[396]
Meriva (Thorne Research Inc., Dover, ID, USA; curcuminoid, 100 mg/day), 60 days (between the 4th and 16th weeks from surgery)	Oncological patients treated with chemotherapy or radiotherapy	↓Side effects	[397]
Micronised curcumin powder (2 g or 4 g per day) 30 days	Subjects with a smoking history (3 years or more)	↓Aberrant crypt foci (40% reduction, only 4 g), 0PGE2, 05-HETE, 0Ki-67	[398]
Curcumin (360 mg/day, capsule), 20 days	Colorectal cancer patients	Tumour tissue ↑p53, ↑Bax, ↓Bcl-2, and ↑cell apoptosis; serum ↓TNF-α; and ↑body weight	[399]
Isoflavones (40 mg/day, 66% daidzein, 24% glycitin, and 10% genistin) and curcumin (100 md) 6 months	Prostate cancer patients	↓PSA (patients with serum level higher 10 ng/mL)	[400]
Polyphenol combination (curcumin 150 mg, resveratrol 75 mg, EGCG 150 mg and soy isoflavone 125 mg each day), 2 weeks	Healthy human volunteers	↓TNF-α activation NF-kB in lymphocytes	[401]
Cur plus capsules (Indsaff LTD, Punjab, India; curcumin 475 mg, piperidin 25 mg)	Subjects with chronic arsenic exposure	↓ROS, ↓DNA damage, ↓lipid peroxidation, ↓protein carbonyl context and ↑antioxidant capacity (↑CAT, ↑SOD and ↓GSH)	[402]
Oxy-Q tablets (Farr Laboratories, Santa Clarita, CA, USA; curcumin 480 mg and quercetin 20 each day), 6 moths	FAP patients	↓Polyp number (~half) and size (~third)	[403]
Curcumin tablet (500–8000 mg, gradually increased each day), 6 months	Patients with a high risk of premalignant lesions	Histological improvement in high-risk premalignant lesions	[404]
Curcumin (6 g/d for 7 days every 3 weeks), 6 months	mCRPC patients treated with docetaxel therapy	No positive results	[405]
CurcuRouge^TM^ (Robertet, Grasse, France; 2 × 90 curcumin mg/d), 4 weeks	Elderly subjects (age > 60 years) with higher NLR	↓Neutrophil count and ratios, ↑lymphocyte ratios, ↓neutrophil/lymphocyte ratio, ↓eosinophil count and ratios	[406]
Nanoparticle curcumin powder (3 × curcumin 1 g/10 mL olive oil per day), 7 days, mouthwash without drinking	Radiotherapy patients with squamous cell carcinoma of the head and neck	↓Radiation-induced oral mucositis	[407]
Meriva (Thorne Research Inc., Dover, ID, USA; curcuminoid, 50 mg/day), 8 weeks	NAFLD patients	↓Mismatched base pairs in DNA and ↓methylation in the MLH1 and MSH2 promoters	[408]
Green tea extract (800 mg/day, topical and systematic) and curcumin (950 mg/day, topical and systematic), 3 months	OPMD patients	Tumour tissue: ↓p53 (mutant type), ↓Ki67, and ↓cyclin D1 (synergic effect)	[409]
APG-157 (Aveta Biomics, Bedford, MA, USA), capsule (3 × 100 or 3 × 200 mg, unfractionated turmeric extract)	Patients with oral cancer	Saliva: ↓IL-1β, ↓IL-6, and ↓IL-8; tumour tissue: ↑CD4+ T cells and ↑CD8+ T cells	[410]
Capsule (500 mg curcuminoid and 5 mg piperine), 8 weeks	NAFLD patients	↓TNF-α, ↓MCP-1, ↓EGF, the cytokine level did not change and ↑NAFLD severity	[411]
SinaCurcumin^®^80 (Nanotechnology ResearchCenter, Mashhad University of Medical Sciences, Mashhad Iran; 80 mg nanoformulation of curcumin/day), 42 days	HNC patients treated with radiotherapy	↓Oral mucositis (one-third) without obvious oral and systemic side effects	[412]
Meriva^®^ (Thorne Research Inc., Dover, ID, USA; curcuminoid, 4 × 100 mg), 28 days	Pancreatic cancer patients treated with gemcitabine therapy	↑OS (10.2 vs. 6.7 month), 0toxicity	[413]
BCM95 (3 × 1.2 g/day, curcumin), 6 months	Patients with oral leukoplakia	↑Clinical and histologic response	[414]
3 × 15 mL EGCG solution (440 μM/L physiological solution), 6 weeks after radiotherapy	Chemotherapy and radiotherapy patients with oesophageal cancers	↓ARIE and ↓RTOG score	[415]
3 × 15 mL EGCG solution (440 μM/L physiological solution), 5 weeks after radiotherapy	Chemotherapy and radiotherapy patients with stage III lung cancer	↑Response rate, ↓pain score	[219]
2 × 10 mL EGCG (μM/L physiological solution), 5 weeks	Chemoradiotherapy patients with stage III NSCLC or limited-stage small-cell lung cancer	↓Maximum oesophagitis grade, ↓pain score and ↓dysphagia score	[131]
Green tea extract (800 mg EGCG/day), 6 weeks	Subjects with a high risk of colorectal cancer	↓NF-kB and ↓DNMT1	[416]
Polyphenon E™ (Mitsui Norin Co., Ltd., Shizuoka, Japan; 400 mg EGCG/day), 12 months	Subjects with a high risk of prostate cancer	↓ HGPIN, ↓ ASAP and ↓PSA	[417]
Polyphenon E ((Polyphenon E International, Inc., NY, USA; 2 × 2000 mg EGCG/day) administered twice daily, 6 months	CLL patients (early-stage)	↓Absolute lymphocyte count and ↓lymphadenopathy	[418]
Six capsules of green tea extract (189 mg EGCG and 97.5 mg caffeine), 6 months	CLL patients	↓Lymphocytosis, ↓absolute number of circulating Treg cells, ↓IL-l0 and ↓TGF-β	[419]
2.5% *w*/*w* EGCG in a silicone in water emulsion, 6 weeks	Healthy volunteers with significant erythema and telangiectasia on the face	↓VEGF and ↓HIF-1α	[420]
Polyphenon E™ (Mitsui Norin Co., Ltd., Shizuoka, Japan; 800 mg EGCG/day), 6 weeks	Prostate cancer patients (stage I-III)	↓HGF, ↓VEGF, ↓PSA, ↓IGF-I, -↓IGFBP-3 and ↓IGF-I/IGFBP-3	[421]
Isoquercetin (500 mg (A), or 1000 (B) mg/day), 56 days	Cancer patients (with pancreatic cancer, NSCLC or colorectal malignancies) at high risk for thrombosis	↓Extracellular protein disulfide isomerase activity (median decrease in D-dimer +9.9 (A), −22% (B)), ↓P selectin (−0.3 (A), −58% (B)) and ↓platelet-dependent thrombin generation	[422]
Quercetin (2000 mg/day), 1 day	Symptomatic sarcoidosis patients	↑TEAC ↓ MDA, ↓TNF-α and ↓IL-8	[423]
Novusetin™ (Bioriginal, Anaheim, CA, USA; fisetin, 100 mg/day), 7 weeks	CRC patients (stage II-III) treated with chemotherapy	↓IL-8 and ↓Hs-CRP	[424]

ARIE, acute radiation-induced oesophagitis; ASAP, atypical small acinar proliferation; Bax, Bcl-2-associated X protein; Bcl-2, B cell lymphoma protein 2; CAT, catalase; CGRP, calcitonin gene-related peptide; CLL, chronic lymphocytic leukaemia; DNMT1, DNA (cytosine-5)-methyltransferase 1; EGF, endothelial growing factor; FAP, familial adenomatous polyposis; GSH, non-enzymatic antioxidant-like glutathione; HGF, hepatocyte growth factor; HIF-1α, hypoxia-inducible factor 1α; HGPIN, high-grade prostatic intraepithelial neoplasia; HRQOL, health-related quality of life; IGF-1, insulin-like growth factor-1; IGFBP-3, insulin-like growth factor binding protein 3; IL-1β, interleukin 1 beta; IL-6, interleukin 6; IL-8, interleukin 8; Il-10, interleukin 10; TGF-β, transforming growth factor-β; hs-CRP, high-sensitivity C-reactive protein; MDA, malondialdehyde; MCP-1, monocyte chemotactic protein-1; MLH1, MutL homologue 1; MSH2, mismatch repair MutS homologue 2; NAFLD, non-alcoholic fatty liver disease; NF-κB, nuclear factor kappa-light-chain-enhancer of activated B cells; NLR neutrophil/lymphocyte ratio; OPMD, oral potentially malignant disorders; PGE2, prostaglandin E2; PSA, prostate-specific antigen; ROS, reactive oxygen species; RTOG, Radiation Therapy Oncology Group; SOD, superoxide dismutase; TEAC, total plasma antioxidant capacity; TNF-α, tumour necrosis factor alpha; VEGF, vascular endothelial growth factor; QoL, quality of life; 5-HETE, 5-hydroxyeicosatetraenoic acid; **↑** = curcuminoids/flavonoids activation/induction; **↓** = curcuminoids/flavonoids repression/inhibition; **0** without change.
